# Trapping and imaging dynamic battery nanointerfaces via electrified cryo-EM

**DOI:** 10.1126/sciadv.adv3191

**Published:** 2025-06-13

**Authors:** Chongzhen Wang, Jung Tae Kim, Xintong Yuan, Jin Koo Kim, Bo Liu, Min-ho Kim, Dingyi Zhao, Matthew Mecklenburg, Yuzhang Li

**Affiliations:** ^1^ Department of Chemical and Biomolecular Engineering, University of California, Los Angeles, Los Angeles, CA 90095, USA.; ^2^ California NanoSystems Institute (CNSI), University of California, Los Angeles, Los Angeles, CA 90095, USA.

## Abstract

The electrified interface between a liquid and a solid underpins diverse phenomena, from ion-transfer during battery operation to action potentials enabling biological communication. However, conventional tools are blind to the nanoscale dynamics of this metastable interface. Here, we leverage electrified cryo–electron microscopy (eCryo-EM), a technique that rapidly freezes and kinetically traps these dynamic, nonequilibrium states during battery operation for nanoscale characterization. Collective snapshots of the electrified interface at controlled time intervals quantifies early-stage growth kinetics of the solid electrolyte interphase (SEI), a passivation film that governs electron and ion transport. Unexpectedly, the diffusivity of charged species of the two SEI films with differing chemistry and performance are estimated to be within 10% of the other, indicated by the slope of their diffusion-limited SEI growth regimes. Instead, the slope of the reaction-limited SEI growth regimes differs by a factor of 3, suggesting that lowered reactivity of the high-performance electrolyte is largely responsible for its high coulombic efficiency.

## Teaser

eCryo-EM quantifies early-stage SEI growth kinetics, revealing reaction-limited growth as key to high electrolyte performance.

## INTRODUCTION

Understanding the dynamic and nonequilibrium processes that occur during battery operation is crucial for accelerating the development of better batteries. Now, these time-dependent and metastable battery states are primarily studied by in situ or operando experiments as ex situ techniques are limited to observing materials in their equilibrium state. Although in situ tools shed light on how electrode morphologies (*1*) and surface chemistries (*2*) evolve during battery operation, important interfacial structures below length scales of ~50 nm are difficult to access, representing a critical challenge for understanding nanoscale processes away from equilibrium (*3*). For example, ion and electron transport between the bulk liquid electrolyte and the anode surface is governed by the solid electrolyte interphase (SEI), a nanoscale interfacial layer whose formation kinetics are poorly understood but strongly dictates next-generation battery technologies based on metallic lithium (*4*–*6*). Thus, accurately resolving the dynamic SEI nanostructure and quantifying its thickness evolution while the battery is operating would be important for our understanding of how Li metal is passivated and reversibly deposited in various electrolyte chemistries. However, such an understanding remains elusive, making the SEI one of the most important yet least understood aspects of battery science (*7*).

Although nonequilibrium stages of SEI formation have been previously explored using in situ liquid phase transmission electron microscopy (TEM), the severe electron beam sensitivity of battery electrolytes and Li metal makes accurate SEI thickness measurements during battery operation challenging. Literature values span almost two orders of magnitude (from 10 to 400 nm), with no clear consensus for the early-stage SEI thickness or its growth rate that are both critical in determining overall battery performance (*8*–*13*). Other surface-sensitive in situ techniques (e.g., x-ray reflectivity, neutron reflectometry, and atomic force microscopy) lack sufficient time and spatial resolution needed to capture early-stage SEI formation on metallic Li (*14*) and are often limited to flat, non-lithium substrates. Thus far, direct imaging using cryo–electron microscopy (cryo-EM) has provided the most consistent measurements of SEI thickness due to increased beam tolerance at cryogenic temperatures (*15*–*21*). However, cryo-EM is inherently an ex situ technique limited to equilibrium-state studies that cannot capture dynamic SEI formation processes while the battery is operating (*22*). As a result, these trade-offs between the ability to image at high spatial resolution (ex situ techniques) and the ability to track dynamic states (in situ techniques) leave key gaps in our understanding of how an SEI film initially forms and its impact on battery operation. Although recent efforts to address these long-standing questions through direct freezing of a coin cell during operation have been proposed, long freezing times (>3 s) and uncontrolled sample thickness result in similar resolution limitations as that of conventional liquid phase TEM, making nanoscale imaging of dynamic SEI formation infeasible (*23*, *24*).

**Fig. 1.**
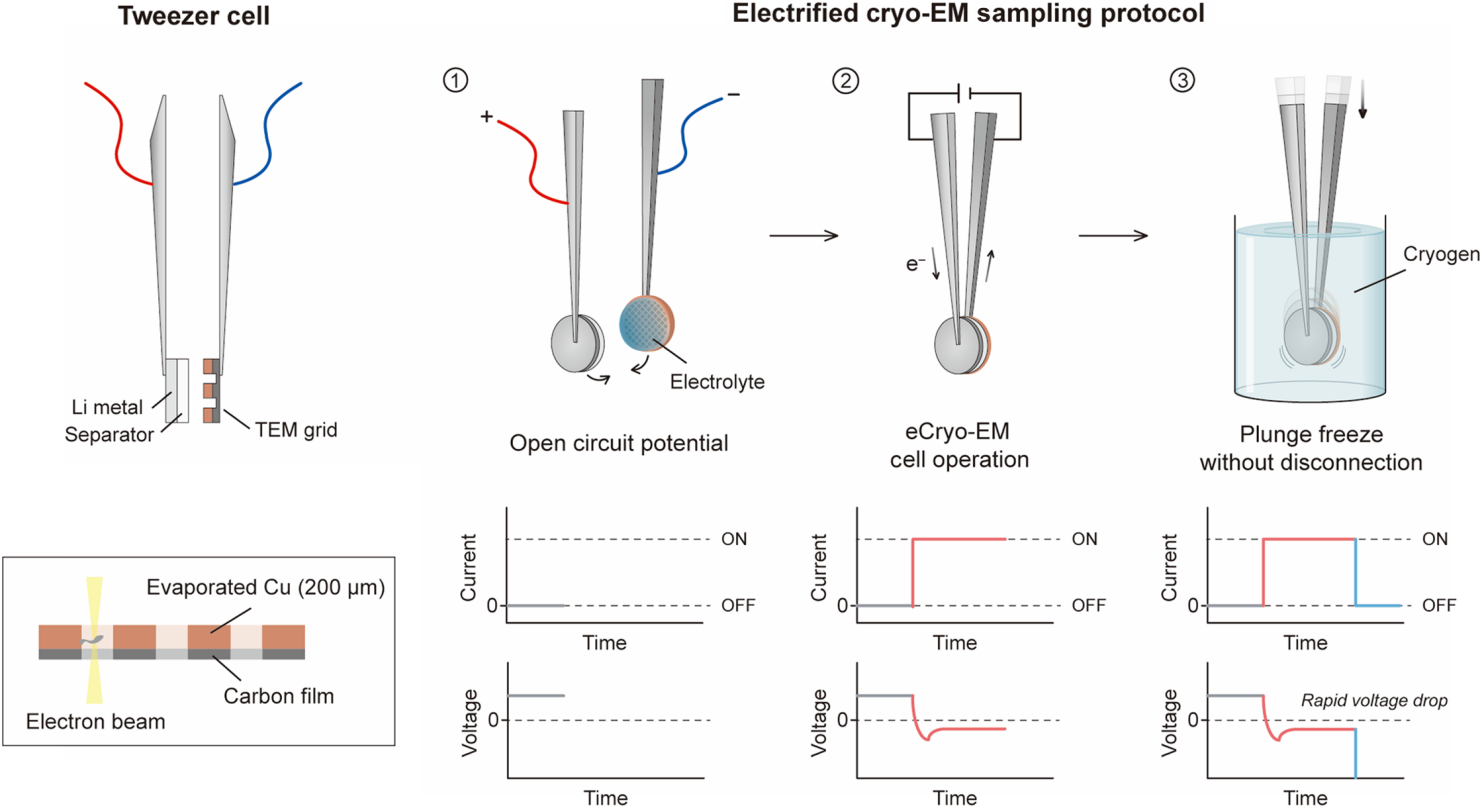
Schematic diagrams illustrating the electrochemical tweezer cell design and eCryo-EM sample preparation procedure. (1) An electrochemical cell is assembled at the tip of a tweezer, allowing constant current to be applied directly for Li deposition on the TEM grid. (2) Without disconnecting the external voltage, the entire tweezer cell is plunged into liquid propane inside an argon-filled glove box, kinetically trapping the dynamic states. (3) The TEM grid is carefully exfoliated from the tweezer cell and then cryo-transferred into the electron microscope without any air exposure.

Here, we develop an electrified cryo-EM (eCryo-EM) technique that enables nanoscale imaging of dynamic processes that are kinetically trapped during air-free battery operation (Fig. 1). The key advantage of eCryo-EM is to rapidly freeze electrochemical reactions while they occur by plunging a thin electrochemical cell directly into cryogen without disconnecting the externally applied voltage. Consequently, dynamic battery processes are cryogenically trapped in their metastable state and cannot relax back to equilibrium, making eCryo-EM distinct from all conventional cryo-EM approaches in the past. The dynamic behavior of the system is then collectively described by eCryo-EM snapshots of these frozen metastable states preserved at various time intervals. This approach quantifies SEI growth kinetics of two model electrolyte systems with differing battery performance, revealing two distinct growth regimes. Initially at early timescales, the linear dependence of SEI thickness with time suggests a reaction-limited regime, with the high-performance model electrolyte exhibiting three times slower growth rate. After ~100 s of Li deposition, the SEI thickness increases with the square root of time, indicating a diffusion-limited regime. Unexpectedly, the diffusion coefficient of charged species (quantified by the slope of diffusion-limited regime) through SEI films formed in both model electrolytes are within 10% of each other, indicating that these chemically distinct SEI films exhibit similar transport properties. These findings highlight the importance of quantifying both electrolyte reactivity and corresponding SEI transport properties for engineering improved battery performance.

## RESULTS

### Tweezer cell fabrication and representativity

To prepare eCryo-EM samples, we first engineer a thin “tweezer cell” that enables rapid freezing (fig. S1). The tweezer cell’s two tips are electrically isolated, with one tip in contact with a Cu TEM grid (working electrode) and the other tip in contact with metallic Li (counter electrode). A commercial battery separator with ~3 μl of liquid electrolyte is sandwiched between the Li metal and Cu TEM grid, completing the electrochemical tweezer cell and allowing current or voltage to be applied across the two electrodes. This tweezer cell design builds on our previous work (*25*) by (i) enabling air-free battery operation and rapid quenching within a glove box and (ii) eliminating physical blotting processes that may otherwise influence the electrochemistry during Li electrodeposition.

**Fig. 2.**
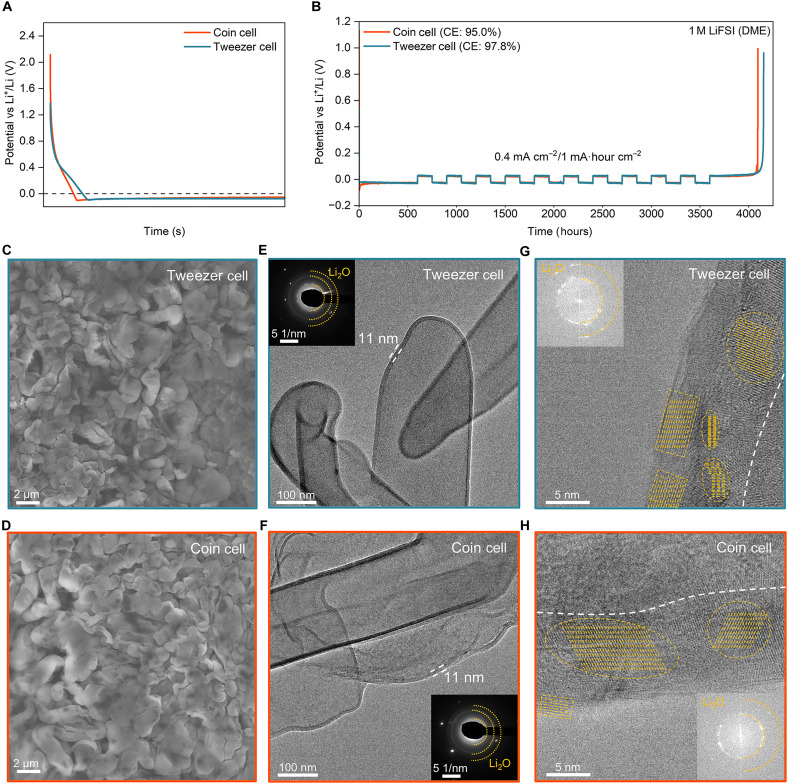
Demonstrating the representativity of the tweezer cell geometry in comparison to coin cell. (**A**) Deposition voltage profile of the tweezer cell (blue) versus a conventional coin cell (orange). (**B**) Galvanostatic cycling of the tweezer cell (blue) and coin cell (orange) at 0.4 mA cm^−2^ and 1.0 mA·hour cm^−2^ in 1 M LiFSI in DME measured using the Aurbach method. (**C** and **D**) SEM images of Li deposited in tweezer cell and coin cell, respectively, at 1 mA cm^−2^ and 0.5 mA·hour cm^−2^ in 1 M LiFSI in DME. (**E** and **G**) Conventional cryo-EM images of Li deposited at 1 mA cm^−2^ and 0.1 mA·hour cm^−2^ and in 1 M LiFSI in DME using the tweezer cell. (**F** and **H**) Conventional cryo-EM images of Li deposited at 1 mA cm^−2^ and 0.1 mA·hour cm^−2^ in 1 M LiFSI in DME using a coin cell. Insets: Corresponding selected-area electron diffraction and FFT images. The yellow dashed circles indicate the crystalline regions of Li_2_O within the SEI.

To demonstrate the reliability of our tweezer cell design, we first compare its electrochemical behavior with that of a typical coin cell. Figure 2A shows that the deposition profiles for the tweezer cell (blue) and coin cell (orange) configurations demonstrate similar electrochemical behavior, including nucleation overpotential and polarization. Similarly, we observe comparable coulombic efficiency (CE) for both configurations during galvanostatic cycling measured using the Aurbach method (Fig. 2B) (*26*). Scanning electron microscopy (SEM) further confirms that the morphology of Li metal electrodeposited in the tweezer cell is identical to that in the coin cell (Fig. 2, C and D). In addition, “conventional cryo-EM” (see Materials and Methods) analysis shows that the SEI on Li metal in both tweezer and coin cell geometries are identical in thickness, nanostructure, and chemical composition (Fig. 2, E to H, and fig. S2). Together, these findings suggest that the observations made in the tweezer cell closely represent those observed in the coin cell, strengthening the representativity of the tweezer cell configuration and eCryo-EM technique demonstrated in our work.

### eCryo-EM sample preparation and imaging

To prepare an eCryo-EM sample, we plunge freeze the entire tweezer cell into liquid propane during Li deposition without disconnecting the circuit. The corresponding tweezer cell voltage immediately plummets to the safety limit of the potentiostat, which occurs simultaneously with the current dropping to zero due to rapid electrolyte vitrification (fig. S3). This voltage and current response indicate that the plunge freezing step (<40 ms) arrests and kinetically traps electrochemical reactions in their dynamic operating state. Heat transfer calculations demonstrate that the freezing timescale of the tweezer stack is in alignment with our experimental result of ~40 ms (fig. S4). Although the tweezer cell is still immersed in the liquid propane, we mechanically exfoliate the TEM grid for subsequent cryo-transfer into the microscope column and eCryo-EM characterization. This minimalistic sample preparation avoids any blotting (*17*), sectioning (*18*), rinsing (*15*), or drying (*20*) steps, which allows us to accurately investigate local structural changes of electrified liquid-solid interfaces as they evolve during electrochemistry (*27*).

**Fig. 3.**
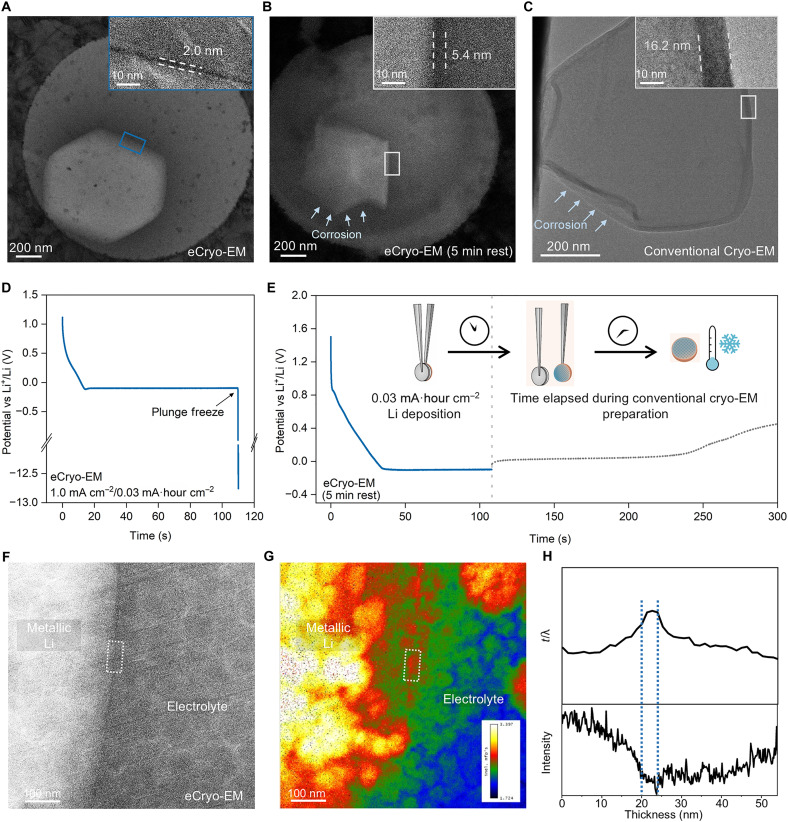
Distinction between eCryo-EM and past conventional Cryo-EM approaches. (**A**) eCryo-EM image of lithium metal deposited via the tweezer cell at 1 mA cm^−2^ and 0.03 mA·hour cm^−2^ in 4 M LiFSI in DME. (**B**) eCryo-EM image of lithium metal deposited via the tweezer cell at 1 mA cm^−2^ and 0.03 mA·hour cm^−2^ in 4 M LiFSI in DME plunge frozen into cryogen after 5-min rest at open circuit voltage (OCV). (**C**) Conventional Cryo-EM image of lithium metal deposited in coin cell at 1 mA cm^−2^ and 0.03 mA·hour cm^−2^ in 4 M LiFSI in DME. (**D**) Voltage profile during eCryo-EM sample preparation. After the tweezer cell is plunged into the cryogen, the voltage rapidly hits the cutoff voltage and current drops to zero. (**E**) Voltage profile during eCryo-EM sample preparation after 5-min rest at OCV. (**F**) eCryo-EM image of Li particle deposited at 1 mA cm^−2^ and 0.1 mA·hour cm^−2^ in 4 M LiFSI in DME. (**G**) Energy-filtered EELS spectrum image and relative sample thickness mapping. The energy loss range is [45 eV, 75 eV]. (**H**) Line and *t*/λ profile from the white dashed box drawn in [(F) and (G)], respectively.

Figure 3A shows a typical eCryo-EM image of an early-stage Li deposit plunge frozen at ~100 s during 1 mA cm^−2^ galvanostatic deposition in a model electrolyte [4 M bis(fluorosulfonyl)imide (LiFSI) in 1,2-dimethoxyethane (DME); ~0.03 mA·hour cm^−2^]. The corresponding voltage profile is shown in Fig. 3D. The amorphous diffraction pattern of the frozen electrolyte (fig. S5) suggests that plunge freezing is sufficiently fast to prevent freezing artifacts (*17*) and preserve the dynamic interfaces of interest (i.e., no additional SEI will form during the rapid freezing process). Because of its lower atomic number, the Li deposit appears to have lighter contrast than that of the vitrified electrolyte, an observation consistent with a previous work (*17*). On the surface of the Li deposit kinetically trapped with eCryo-EM, we find the presence of an ultrathin SEI layer (~2 nm), a finding that has not been observed before with conventional techniques. This ultrathin SEI is much darker in contrast compared to both the Li metal and the vitrified electrolyte, indicating a highly dense and inorganic structure.

The ultrathin nature of the early-stage SEI suggests that poor passivation of initial Li deposits is the reason for their observed dissolution and corrosion at open circuit potential (Fig. 3E). For example, Li deposits imaged using eCryo-EM (Fig. 3A) exhibits a smooth and rounded morphology. However, Li deposits appear to be rough and pitted in a control experiment (Fig. 3B), where tweezer cell samples with the same deposition capacity are allowed to rest at open circuit voltage (i.e., no current being applied) for ~5 min prior to plunge freezing. This irregular morphology and the gradually increasing open circuit voltage during rest suggest that Li metal at low deposition capacity (<0.03 mA·hour cm^−2^) is not sufficiently passivated and highly susceptible to self-discharge (*28*) or corrosion during even short rest times at open circuit voltage (*29*). It is important to note that the 5-min rest condition is unavoidable during battery disassembly and sample preparation required for conventional techniques. Conventional cryo-EM imaging shows that the SEI layer on low-capacity Li (0.03 mA·hour cm^−2^) is five times as thick as that on kinetically trapped Li imaged with eCryo-EM at the same capacity (Fig. 3C and fig. S6), an experimental artifact likely caused by chemical corrosion (*30*) and a potential limitation of conventional cryo-EM to investigate early stage Li nuclei and its SEI film. Instead, eCryo-EM arrests all electrochemical reactions (including corrosion) during plunge freezing, kinetically trapping metastable states for accurate analysis.

Chemical mapping via electron energy-loss spectroscopy (EELS) in conjunction with eCryo-EM further confirms that the thin dark layer we imaged corresponds to the SEI rather than any imaging artifacts (Fig. 3, F and G, and fig. S7). We consistently observe a spike in the *t*/λ profile, coinciding with the thin dark layer in the corresponding eCryo-EM image and energy-filtered EELS spectrum image (Fig. 3H). This peak in the *t*/λ profile originates either from an increase in the local nanoscale thickness of our sample (*t*) in the *z* direction or an increase in the local density (a decrease in the mean free path, λ) (*31*). Because it is extremely unlikely to have a sudden nanoscale change in the sample thickness (e.g., a <10-nm protrusion in the *z* direction) within the relative uniform sample thickness in surrounding regions (especially directly coinciding with the dark SEI region), we interpret this sharp peak as an increase in the local sample density. This directly coincides with the dark contrast region hypothesized to be the SEI, which is expected to be much denser than metallic Li or liquid electrolytes for the enrichment with inorganic species. Furthermore, eCryo-STEM EELS mapping of the Li K edge indicates that the thin dark layer we observe is composed of inorganic species such as Li_2_O and LiOH, which coincides well with typical SEI components (fig. S8) (*32*). Last, additional EELS experiments demonstrate that variations in the sample thickness in the *z* direction (ranged from 300 to 600 nm; assuming a mean free path of 200 to 250 nm) (*31*) do not markedly affect our SEI thickness measurements (fig. S9).

### Quantifying early-stage SEI growth kinetics via eCryo-EM

**Fig. 4.**
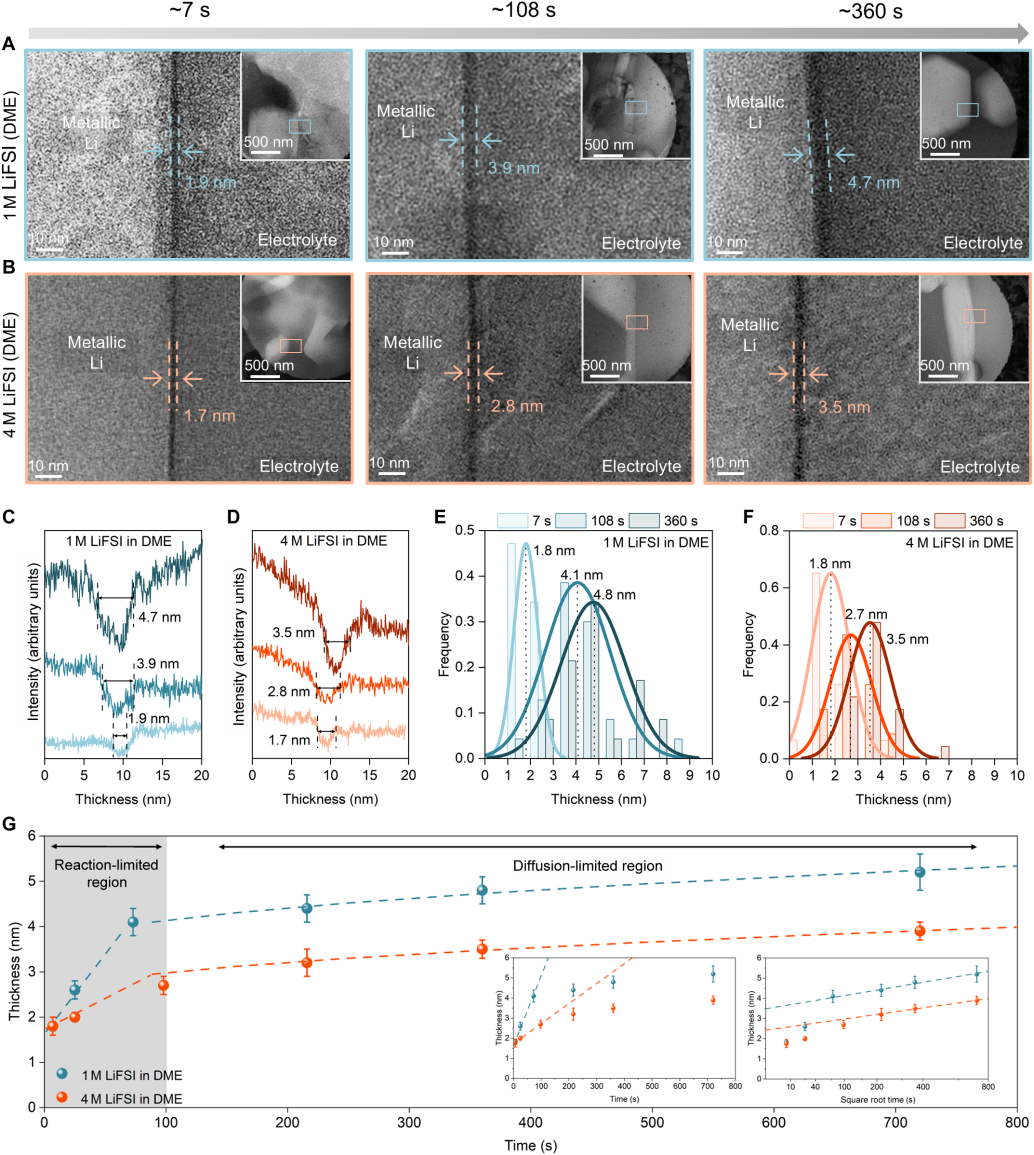
Growth kinetics of early-stage SEI revealed by eCryo-EM. (**A** and **B**) eCryo-EM images of lithium deposited in 1 M LiFSI in DME and 4 M LiFSI in DME. The capacity is ~0.002, 0.03, and 0.1 mA·hour cm^−2^ from left to right. (**C** and **D**) Line profiles of image intensity (arbitrary units) across the SEI in 1 M LiFSI in DME and 4 M LiFSI in DME. Profiles offset from bottom to top correspond to increasing deposition capacity at increasing time intervals of plunge freezing. (**E** and **F**) Distribution of SEI thickness under different deposition capacity in 1 M LiFSI in DME and 4 M LiFSI in DME. Curve is the Gaussian distribution. (**G**) SEI thickness plot versus lithium deposition time, defined as time spent below 0 V versus Li metal in the electrochemical profiles. Inset (left) plots SEI thickness versus a linear *x* axis. The first three data points appear linear with time, indicating a reaction-limited growth regime. Inset (right) plots SEI thickness versus the square root of time. The last three data points appear linear to the square root of time, indicating a diffusion-limited growth mechanism. The slopes for both the high-performance and conventional electrolyte are within 10% of each other in the diffusion-limited regime.

To accurately monitor how the SEI evolves at early timescales, we plunge freeze the electrochemical tweezer cell at controlled time points during electrodeposition using eCryo-EM. Figure 4G plots a time series of the SEI thickness measured at various points during electrodeposition. The voltage profiles for all the data points are shown in fig. S22. At least 15 independent regions are averaged for each time point to ensure consistency and accuracy (Fig. 4, C to F, and figs. S10 to S23). The growth plot reveals two distinct SEI growth regimes during electrodeposition: (i) a reaction-limited regime followed by (ii) a diffusion-limited regime. Initial SEI thickness appears to be independent of electrolyte chemistry, forming an ultrathin SEI (~1.7 nm) after 7 s of electrodeposition in both a conventional (i.e., 1 M LiFSI in DME) and high-performance (i.e., 4 M LiFSI in DME) electrolyte (Fig. 4, A and B). This suggests that the difference in SEI chemistry (i.e., solvent derived versus anion derived) does not affect initial SEI formation, which occurs if transport through the SEI is sufficiently fast to keep pace with the rapid reaction kinetics between Li and electrolyte (*33*). This may suggest that electron tunneling processes are present at these ultrathin length scales (*34*), where passivation properties of the SEI composition do not play a considerable role yet. In other words, the SEI cannot slow down the Li metal reaction with electrolyte at ultrathin length scales (<1.7 nm), which also explains why Li metal is susceptible to chemical corrosion at early timescales before sufficient passivation (*35*). Beyond this initial ultrathin length scale, the SEI thickness grows linearly with time within the reaction-limited regime, the slope of which is a quantitative metric of reactivity. We find that the high-performance electrolyte (4 M LiFSI in DME) exhibits a slope 3x lower than that of the conventional electrolyte (1 M LiFSI in DME), indicating reduced reactivity of the 4 M LiFSI in DME with metallic Li. This finding is consistent with the electrode potential measured for such electrolytes, which is higher (i.e., less reactive) for 4 M LiFSI in DME (*36*).

At later timescales (>100 s), SEI growth transitions to a diffusion-limited regime, which is indicated by the square root dependence of SEI thickness with time (Fig. 4G and fig. S21). Correspondingly, the slope of the SEI thickness versus $\sqrt{t}$ profile is proportional to the diffusivity of the diffusion-limiting species (*30*, *37*, *38*). Unexpectedly, the slopes of both the high-performance and conventional electrolyte are within 10% of each other, suggesting that transport through these two SEI films are quantitatively similar. This finding challenges the conventional wisdom that suggests anion-derived SEI films (e.g., formed in 4 M LiFSI in DME) are much more “passivating” than solvent-derived SEI films (e.g., formed in 1 M LiFSI in DME). Although it is presently unclear what the diffusion-limiting species through the SEI is (e.g., either electrons diffusing to the SEI-electrolyte interface or anions/solvents diffusing to the Li-SEI interface), the SEI growth profiles suggest that diffusion-limited growth (most Li metal deposition and battery operation) proceeds similarly for these two distinct SEI chemistries.

The key distinction between high-performance and conventional electrolyte SEI films appears to be their initial reactivity rather than their transport properties. Reduced reactivity between Li and the electrolyte can enhance performance in two ways: (i) by limiting SEI formation that otherwise reduces CE and (ii) forming a thinner SEI layer facilitates more efficient Li-ion transport while effectively passivating metallic lithium, reducing dendrite formation, and enhancing overall battery performance. Together, the SEI growth kinetics revealed by eCryo-EM suggest that decreasing reactivity of Li metal is an equally important yet underexplored design strategy compared with efforts in SEI engineering.

## DISCUSSION

In conclusion, we have established eCryo-EM as a unique tool capable of trapping and imaging dynamic processes for nanoscale imaging, which conventional techniques are blind to. This allows us to uncover the SEI growth kinetics on metallic Li, which has remained elusive due to their high reactivity. Unexpectedly, we find that the transport properties through the SEI of both high-performance and conventional electrolytes are within 10% of one another, suggesting similar passivating properties. Instead, their reactivity differs by a factor of 3, which is likely the key reason for enhanced performance. On the basis of these findings, a key design principle to improve battery performance may be to decrease the reactivity of both solvent and salt molecular components. This refines previous semiempirical approaches of fluorinating all electrolyte components or targeting anion-derived SEI films, opening an underexplored design space for improving battery performance. More broadly, eCryo-EM provides a general approach to study the dynamic nanoscale processes at electrified interfaces fundamental to all electrochemical devices (e.g., electrocatalysts and supercapacitors), introducing exciting opportunities for scientific exploration and discovery. This study establishes a foundation for the next generation of cryo-EM technologies, which may also have broad implications for biology, where electrified interfaces are the basis of facilitating action potentials that enable communication between biological cells.

## MATERIALS AND METHODS

### Materials

Commercial battery separators, composed of polypropylene/polyethylene/polypropylene, with a thickness of 25 μm, were purchased from Celgard (2325). Stainless steel coin cell cases, stainless steel spacers, and springs were purchased from MTI. Battery-grade Li foil (750 μm thick) and copper current collector (25 μm thick) were purchased from Alfa Aesar. J-B Weld 8276 KwikWeld epoxy was purchased from Amazon. The electrochemical tweezer cell was fabricated using PELCO Pro gold-plated tweezers purchased from Ted Pella. Instrument-grade (>99.5%) propane gas was purchased from High Precision Gas. The foam vitrification Dewar, propane cup, metal spider, and grid box ring were purchased from Ted Pella. Holey carbon TEM grids (300 mesh) were purchased from Quantifoil. LiFSI was purchased from Canrd (99.5%). DME was purchased from Sigma-Aldrich (99.5%). Quantifoil R2/2 holey carbon TEM grids with a 200-nm surface layer of copper were used for all eCryo-EM experiments. Copper was deposited onto the Quantifoil grids using an e-beam evaporation process using a KJ Lesker e-gun/beam evaporator, maintaining a vacuum pressure below 1 × 10^−6^ torr. Center-marked copper grids (300 mesh) were purchased from TedPella and were used for all conventional cryo-EM experiments.

### Electrochemical tweezer cell fabrication

Figure S1 shows optical images of the electrochemical tweezer cell and all the necessary components required for its fabrication. To begin, a PELCO 20 tweezer is mechanically separated into two individual pieces using a plier. The bare copper strands of a battery connection cable are carefully exposed from their plastic covering using a wire stripper. The copper strands are then wrapped around the handle of each tweezer piece. Insulating tape is wrapped over the copper strands to fix them in place and to prevent direct contact between the two tweezer pieces. Two 5 mm–by–5 mm stainless steel plates are cut from a 2032-coin cell case using sharp scissors and then welded to the tip of each individual tweezer piece. An electrically insulating printed circuit board (PCB) is fixed in between the two tweezer pieces using J-B weld epoxy. The PCB functions to rejoin the two tweezer pieces such that they still function as a tweezer but with the two tips electrically isolated.

### Electrolyte solution preparation

LiFSI was dried on a hot plate at 80°C for 12 hours in an Ar-filled glove box before use. LiFSI was then added to DME and mixed to obtain a 1 or 4 M solution of LiFSI in DME. All chemicals were used as received without further purification. All electrolytes were made and stored in the argon-filled glove box (oxygen < 0.01 parts per million).

### eCryo-EM sample preparation

To obtain liquid propane, a foam vitrification Dewar equipped with a propane cup, metal spider, and grid box ring was filled to the brim with liquid nitrogen. Propane gas was then piped into the cold cup via a plastic nozzle, allowing the propane gas to condense until the cup was filled with liquid propane. The counter electrode was fabricated by punching commercial Li foil into an ~3.5-mm-diameter disk. Battery-grade Cu foil was cut into a square with an area of 16 mm^2^, serving as the working electrode. A Celgard 2325 separator was cut into a square with an area of ~20 mm^2^ to divide the working and counter electrode. First, 3 μl of electrolyte was deposited onto the surface of a Li disk (3.5 mm in diameter, 0.75 mm thick) using a pipette. The electrolyte volume was selected to ensure good electrical connection while avoiding the formation of a thick electrolyte film unsuitable for TEM imaging. A Celgard separator (~20 mm^2^) was then placed atop the electrolyte-wetted Li disk, followed by the placement of a 200-nm Cu-evaporated Quantifoil TEM grid. Last, a piece of Cu foil (~16 mm^2^) was placed on top of the TEM grid. Special care was given to ensure that all components were centered with one another. It is worth mentioning that the electrolyte stayed at the counter electrode (Li disk) side and no more electrolyte was added after placing the separator. The penetration of electrolyte under capillary action helped form a uniform thin liquid layer in the holes of TEM grids. Once all the components of the cell had been carefully stacked, the stack was delicately placed between the two stainless steel plates of the electrochemical tweezer cell setup and secured using a binder clip. At different time intervals during Li metal deposition (BioLogic VMP3), the tweezer cell was manually submerged into the small cup of liquid propane. All measurements were obtained under a constant current of 1 mA·cm^−2^. The TEM grid was mechanically exfoliated while still submerged in liquid propane to avoid exposure to the ambient environment. Once the TEM grid had been exfoliated, the cup of liquid propane was transferred outside of the glove box such that the submerged TEM grid could be transferred into a cryo-grid box and stored in a large Dewar of liquid nitrogen. It is important to note that no blotting was applied to absorb excess amounts of electrolyte when preparing eCryo-EM samples.

### Coin cell assembly

2032-type coin cells are used for all electrochemical measurements conducted in a coin cell configuration. Li (working electrode) and Cu foil (counter electrode) were punched into 10- and 12-mm-diameter disks, respectively. The Li foil surface was mechanically polished using scrapers to remove the surface oxide layer. A 25-μm Celgard separator was used to separate the two electrodes. An 80-μl electrolyte was added to each cell. Bare copper TEM grids were placed on top of the copper foil during cell assembly for conventional cryo-EM experiments. All cells were crimped at 1000 psi and tested at 25°C using a Biologic VMP3 Potentiostat. A current density of 1 mA cm^−2^ is applied to deposit lithium directly onto the TEM grids under varying time intervals.

### Electrochemical testing

Cycling performance testing was conducted on a BioLogic VMP3. Cu||Li cells were assembled in the tweezer setup and coin cell as the methods described above and were cycled at 4 mA·hour cm^−2^/1 mA·hour cm^−2^/0.4 mA cm^−2^ for 10 cycles.

### Conventional cryo-EM sample preparation

“Conventional” cryo-EM refers to samples that have been preserved in the absence of vitrified electrolyte. Coin cells were disassembled in an Ar-filled glove box, where the Cu TEM grid was retrieved from the working electrode and carefully rinsed with 100 μl of 1,3-dioxolane (DOL) to remove electrolyte residue on the grid. After rinsing, the Li-deposited TEM grid was placed between two Kimtech Kimwipes and manually pressed for ~1 min to remove DOL residue. Once dry, the TEM grid was placed in an Eppendorf tube and tightly sealed using parafilm. The sealed Eppendorf tube was then transferred out of the glove box, immediately submerged into a Styrofoam pot filled with liquid nitrogen, and crushed using a pair of pliers. The submerged TEM grid was placed into a cryo-box and stored in a large Dewar filled with liquid nitrogen for subsequent cryo-EM imaging.

### Cryo-EM sample transfer

TEM grids were mounted onto a TEM cryo-transfer holder (Gatan 626) using a cryo-transfer station to ensure that the entire transfer procedure occurred under liquid nitrogen. A built-in shutter on the holder is closed during sample insertion into the TEM column (~1 s) to prevent the sample from air exposure and ice condensation. Once inserted, a Dewar attached to the holder is filled with liquid nitrogen, which keeps the sample cold at −178°C.

### Electron microscopy

All eCryo-EM characterizations were performed using an FEI Titan 80-300 scanning transmission electron microscope operated at 300 kV equipped with a field-emission gun (X-FEG), Oxford X-MaxN 100TLE 100-mm^2^ SDD x-ray spectrometer, Gatan Ultrascan digital camera, and DE-64 direct detection camera (unless otherwise stated). For all eCryo sample characterization, the fluence is controlled at ~60 e/Å^2^·s to reduce beam effect on the vitrified electrolyte. Low fluence (colloquially called low dose) function in serialEM software was used to further avoid any beam exposure before image capture. The capture time for each TEM image was 0.25 s such that the total dose to obtain one image was 15 e/Å^2^. An objective aperture (20 μm in diameter) was inserted to enhance the thickness contrast and decrease the diffraction contrast. eCryo-EELS was conducted on a JEOL Grand ARM 60-300 kV S/TEM equipped with a cold field-emission gun and configured with a wide gap pole piece (>6 mm), a high-angle annular dark-field STEM detector, a dedicated annular bright-field detector, a direct electron detection system (the Gatan K2 IS/Summit), and a Gatan 965 GIF Quantum ER imaging filter with dual-energy range EELS (DualEELS).

### SEM characterization

SEM images were taken using a ZEISS Supra 40VP SEM with an acceleration voltage of 10 kV. SEM samples were prepared by first rinsing them with a few drops of anhydrous DOL. After drying the samples for ~10 min in argon atmosphere, they were fixed onto an SEM stage using carbon tape. The stage was then placed in a Teflon container and tightly sealed using parafilm. The SEM stage was quickly transferred into the SEM chamber (~5 s) to avoid exposure to the ambient environment.

### TEM data processing

High-magnification eCryo-EM images were fast Fourier transformed in Gatan Microscopy Suite software. A circle (~5 nm^−1^) was selected and excised from the fast Fourier transform (FFT) of the image. Then, inverse FFT was applied to the circle region to mask out detailed information under 0.2 nm and enhance the signal-to-noise ratio. For eCryo-EM images, brightness and contrast were adjusted for better illustration.

EELS data were processed in Gatan Microscopy Suite software. EELS spectrum image was binned by (4,4,2), and the background was subtracted using first-order log-polynomial model. Li K-edge and O K-edge EELS signal counting profiles were extracted from corresponding regions after background subtraction. The multiple linear least squares mapping image was processed by mapping binned spectrum image reference to spectral signature extracted from corresponding regions.
