# Supplementary material for: Trapping and imaging dynamic battery nanointerfaces via electrified cryo-EM
Source: Sci Adv. 2025 Jun 13;11(24):eadv3191. doi: 10.1126/sciadv.adv3191 (PMC12164978; doi:10.1126/sciadv.adv3191)
Supplement: Supplementary file 1 — Figs. S1 to S24 [file sciadv.adv3191_sm.pdf]

Science Advances
AAAS

Supplementary Materials for

Trapping and imaging dynamic battery nanointerfaces via electrified cryo-EM

Chongzhen Wang *et al.*

Corresponding author: Yuzhang Li, yuzhangli@ucla.edu

Sci. Adv. **11**, eadv3191 (2025)
DOI: 10.1126/sciadv.adv3191

This PDF file includes:

Figs. S1 to S24

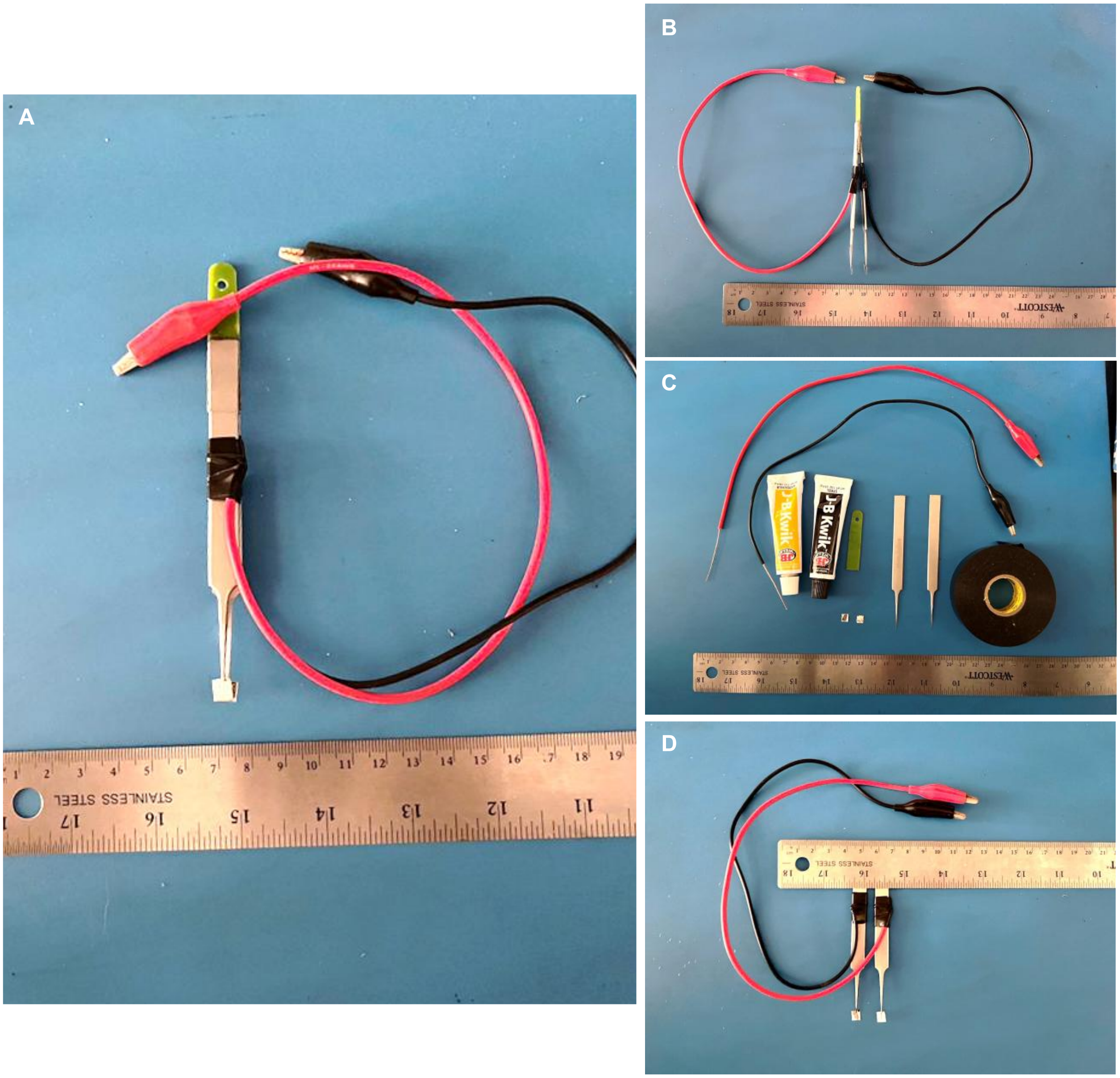

Figure S1. Optical images of the eCryo tweezer cell and its fabrication. Front view (**A**) and side view (**B**) of the eCryo tweezer. (**C**) Two individual tweezer pieces that have been wrapped with copper wires and the tips welded with stainless steel plates. (**D**) All the raw materials used to fabricate the eCryo tweezer.

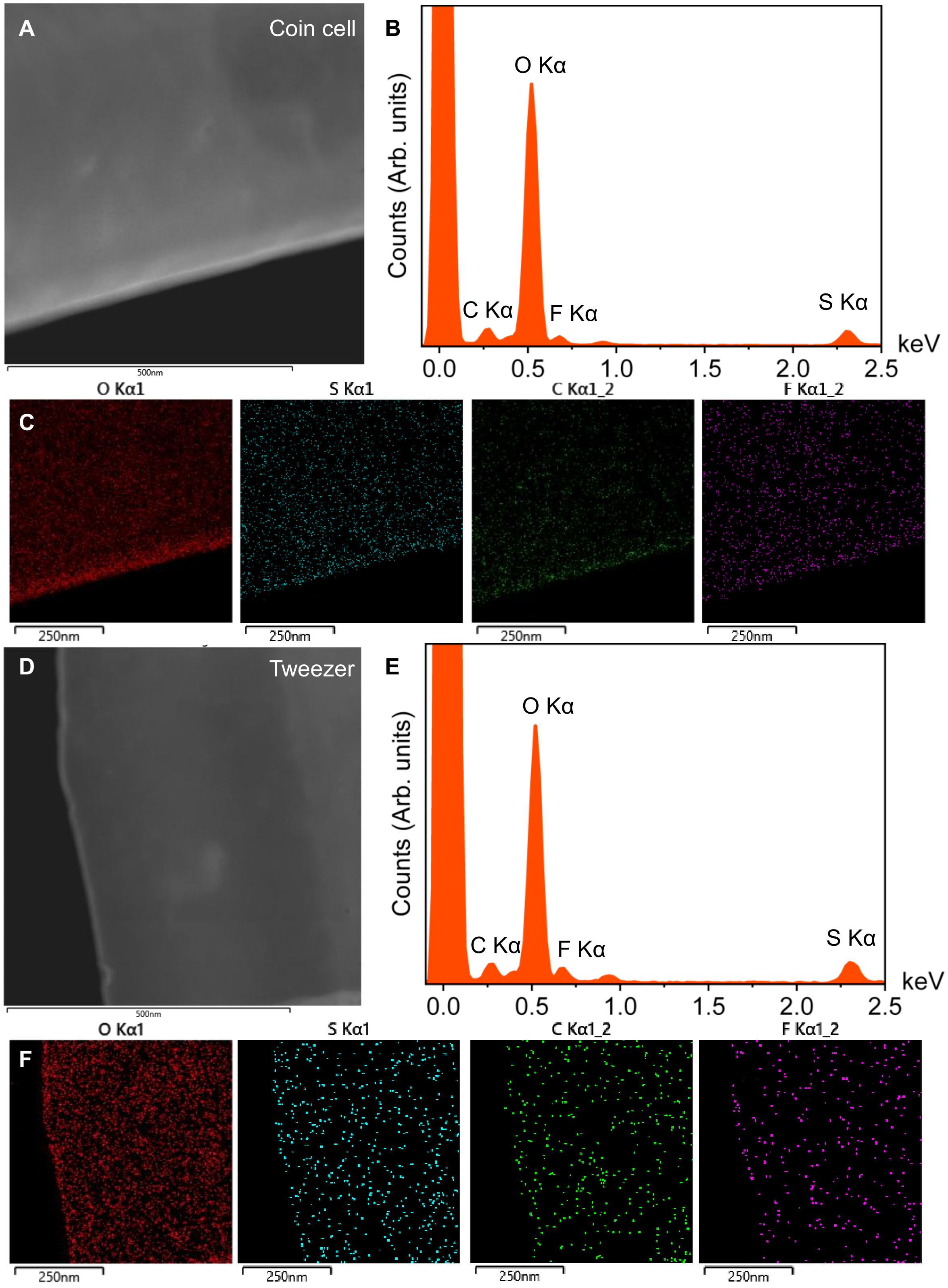

Figure S2. Comparing SEI formed in tweezer cell versus coin cell. (**A** & **D**) Cryo-STEM image of Li deposited at 0.1 mA cm^{-2} and 1 mAh cm^{-2} in 1M LiFSI in DME from (**A**) the coin cell and

(**D**) the tweezer cell. (**B** & **E**) EDS spectrum of metallic Li deposited from the coin cell (**B**) and tweezer cell (**E**). The composition of SEI formed in tweezer cell is nearly indistinguishable from that of the coin cell. (**C** & **F**) EDS mapping of O, S, C, F signal.

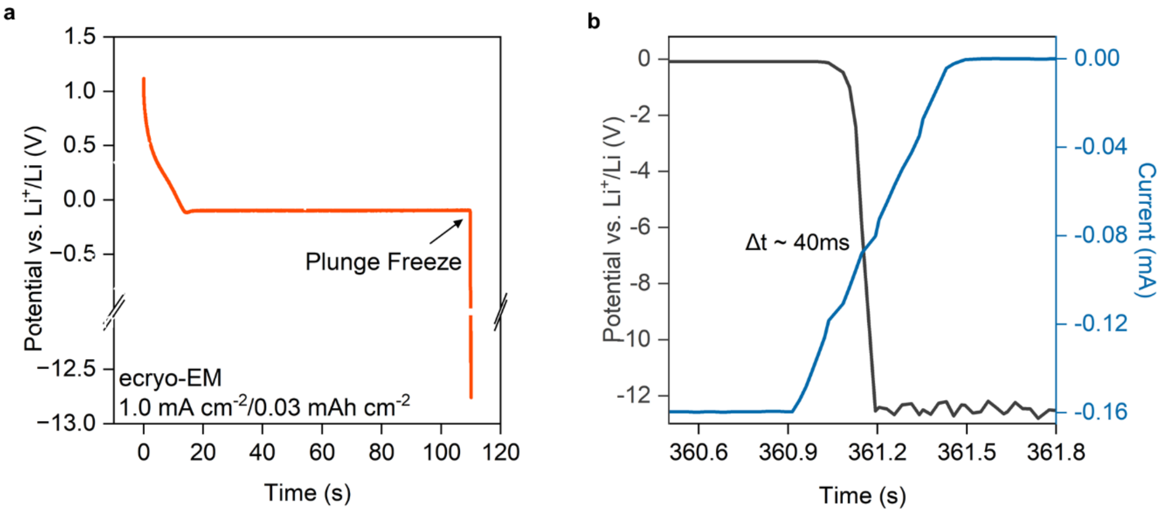

Figure S3. Electrochemical behavior of the tweezer cell during eCryo-EM sample preparation. (**A**) Voltage profile during eCryo-EM sample preparation. After the tweezer cell is plunged into the cryogen, the voltage rapidly hits the cutoff voltage and current drops to zero. (**B**) Current profile showing that vitrification can be achieved within ~40 ms.

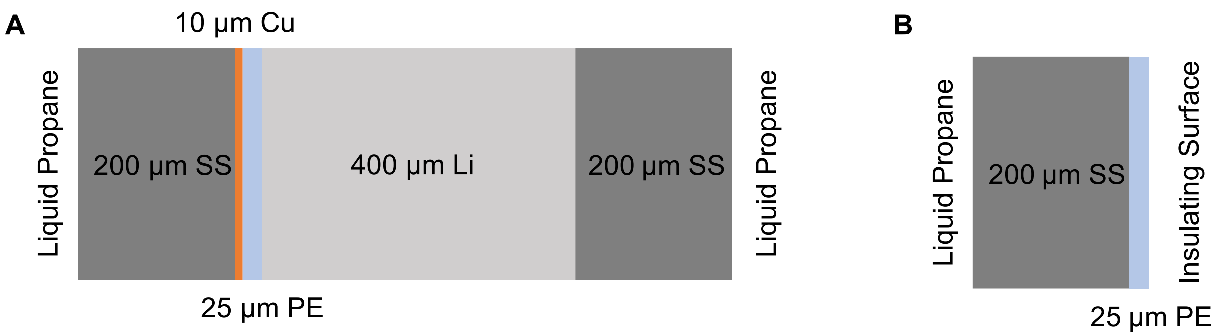

Figure S4. Cooling Rate Calculation. (**A**) Schematic diagram of the entire tweezer cell. (**B**) Schematic diagram of simplified model used for method 2 calculation. The tweezer system can be modeled as a heterogenous slab composed of two 200 μm thick stainless-steel plates, a 400 μm thick Li plate, a 10 μm thick copper foil and a 25 μm thick PE separator with electrolyte. The initial temperature of the slab is uniform at room temperature T_0, which is 25 °C. The temperature of liquid propane is assumed to be T_1 = -150 °C. The heat conduction is assumed to take place through the left and right surface of the slab. For electrochemical reactions to be kinetically trapped, a thin film of the liquid electrolyte (<~3nm; or the electron tunneling length scale) needs to reach the target temperature of its freezing point (e.g., DME, T_melting = -58 °C). This solidification will arrest ion and electron transport across the negative and positive electrode. To calculate the timescale of this solidification, we model the heat transport using two methods below:

Method 1:

Since the whole slab is mainly composed of Li and stainless-steel, we simplify the system by assuming it to be a homogenous 835 μm thick slab with the same thermal properties of Li or stainless-steel, which is

$$\alpha_{Li} = 37\, mm^2 \cdot s^{-1}$$

$\alpha_{SS} = 4\, mm^2 \cdot s^{-1}$,

where α is the heat diffusivity of Li and stainless-steel.

Now our system becomes simplified into a one-dimensional unsteady state heat conduction problem in a homogenous slab facing a heat sink at both sides. Then, the temperature as a function of distance and time T(y, t) follows the equation below; detailed derivations can be found in a classic textbook. (Transport Phenomena, BSL, 2002)

$$\frac{T_1 - T}{T_1 - T_0} = 2\sum_{n=0}^{\infty} \frac{(-1)^n}{\left(n+\frac{1}{2}\right)\pi} \exp\left[-\left(n+\frac{1}{2}\right)^2 \pi^2 \alpha t / b^2\right] \cos\left[(n+\frac{1}{2})\frac{\pi y}{b}\right]$$

where b is equal to half the slab thickness and y is the distance from the center of the slab.

The point of interest is the copper-separator interface, where y/b = 0.497, the dimensionless temperature is derived by

$$\frac{T - T_0}{T_1 - T_0} = \frac{-58 - 25}{-150 - 25} = 0.474$$

By solving the governing equation above, we can calculate the dimensionless time $\alpha t/b^2$, which is approximately 0.3, giving the time t, for electrolyte to freeze as 1.4 ms assuming the slab is lithium (a lower bound estimate) or 13 ms assuming the slab is stainless-steel (an upper bound estimate). Since the real slab is heterogeneous, which has an effective heat diffusivity between that of lithium and stainless-steel, the estimated freezing timescale should be in the range of 25 milliseconds [1.4 ms, 13 ms].

Method 2:

Since the point of interest is the copper-separator interface (where our eCryo-EM imaging eventually occurs), which is much closer to the left part of the slab, it is likely that the electrolyte has reached the freezing temperature while the temperature of the center of the slab is higher than the target temperature. Thus, we can consider the heat conduction for only the left slab surface and ignore the right part of the slab, treating the PE as a thermal insulator. Besides, since the copper is much thinner and the heat diffusivity of copper is much larger than stainless-steel, the influence of copper is assumed negligible. Now the system is simplified into a two-layer slab which is composed of a stainless-steel plate and the separator with electrolyte, and the heat conduction only takes place through the surface of stainless-steel (**Figure S4B**). Ignoring the copper foil, our sample is directly facing the right surface of the stainless-steel, so the freezing time to estimate is the time for the right side of the stainless-steel to reach the target temperature (i.e., freezing point of the electrolyte) and now it becomes a one-dimensional unsteady state heat conduction in a 8 homogeneous slab facing a heat sink at one side. We use y as the distance from the left surface of the slab and the point of interest is at y = 0.2 mm. The temperature function T(y, t) follows the equation, detailed derivations of which can be found in the same classic textbook. (Transport Phenomena, BSL, 2002)

$$\frac{T - T_0}{T_1 - T_0} = 1 - \mathrm{erf}\left(\frac{y}{\sqrt{4\alpha t}}\right)$$

where α is the heat diffusivity of stainless-steel.

By solving the equation, we can estimate that the time for electrolyte to freeze is 39 ms, which is higher than the value derived from Method 1 because we ignore the heat transport from the right part of the system. These calculations are in alignment with our experimentally determined freezing time of ~40 ms.

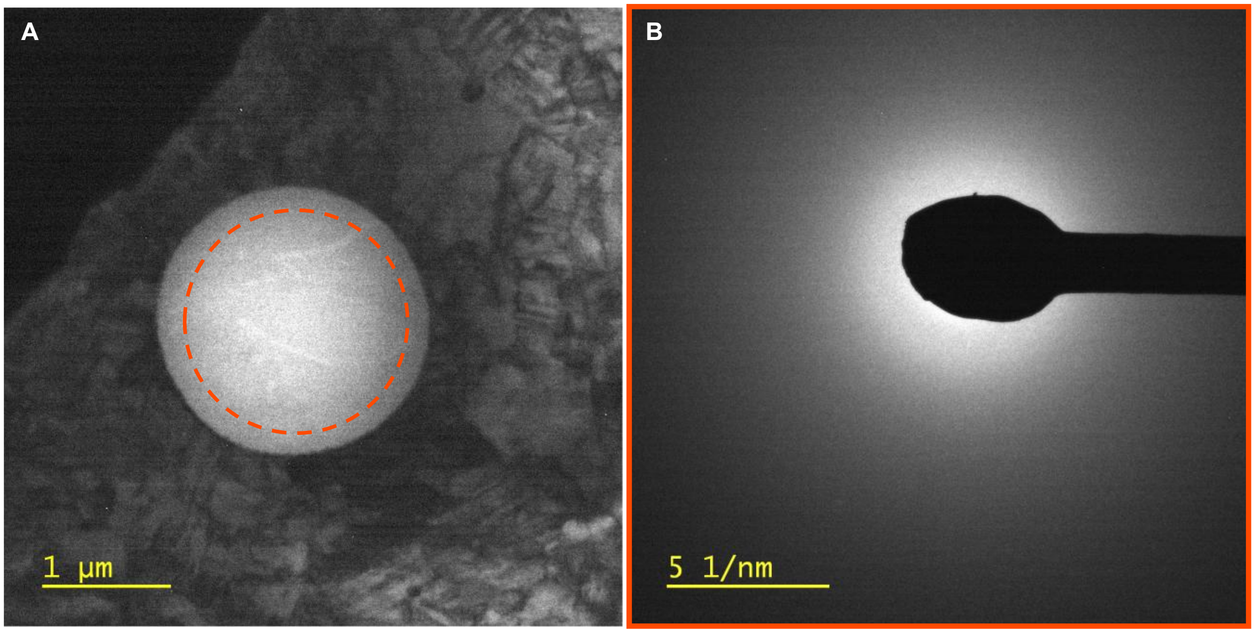

Figure S5. eCryo-EM image and diffraction pattern of vitrified electrolyte. (**A**) A typical eCryoEM image of vitrified electrolyte on 200 nm copper evaporated Quantifoil grid. (**B**) Diffraction pattern of the circled area in the eCryo-EM image. The absence of diffraction pattern indicates that the vitrified electrolyte is amorphous.

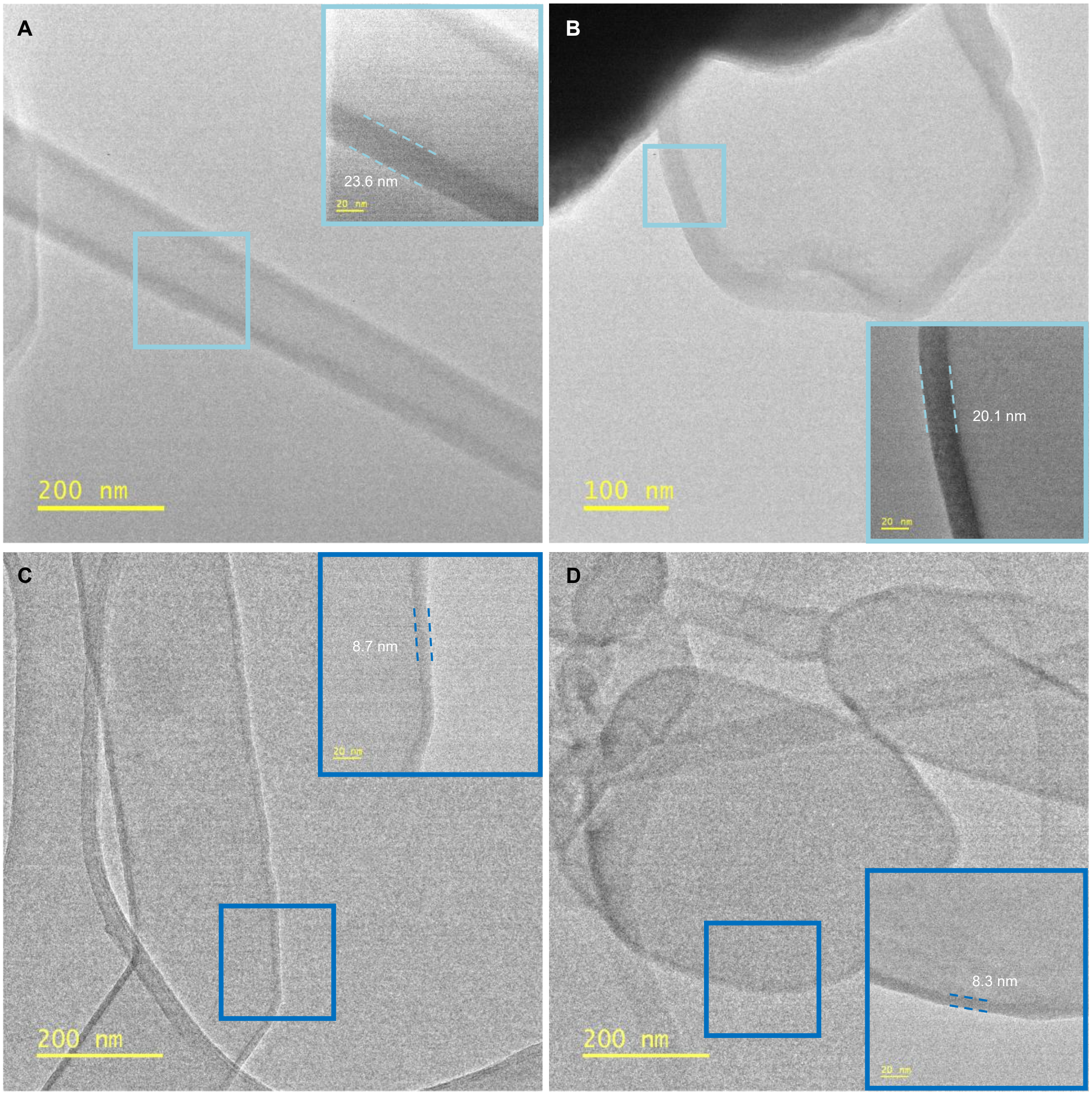

Figure S6. Conventional Cryo TEM images at different capacity. Conventional Cryo TEM images of lithium particles deposited at 1 mA cm^{-2} and 0.01 mAh cm^{-2} (**A** & **B**) and 1 mAh cm^{-2} (**C** & **D**) in 1M LiFSI in DME. The inset images are the zoom-in of the square region in the images. The SEI thickness at 0.01 mAh cm^{-2} is more than doubled compared to that at 1.0 mAh cm^{-2} capacity. At low capacity, early-stage SEI cannot passivate Li particles well, making them more prone to corrosion when compared to lithium particles with more developed SEI deposited at high capacity.

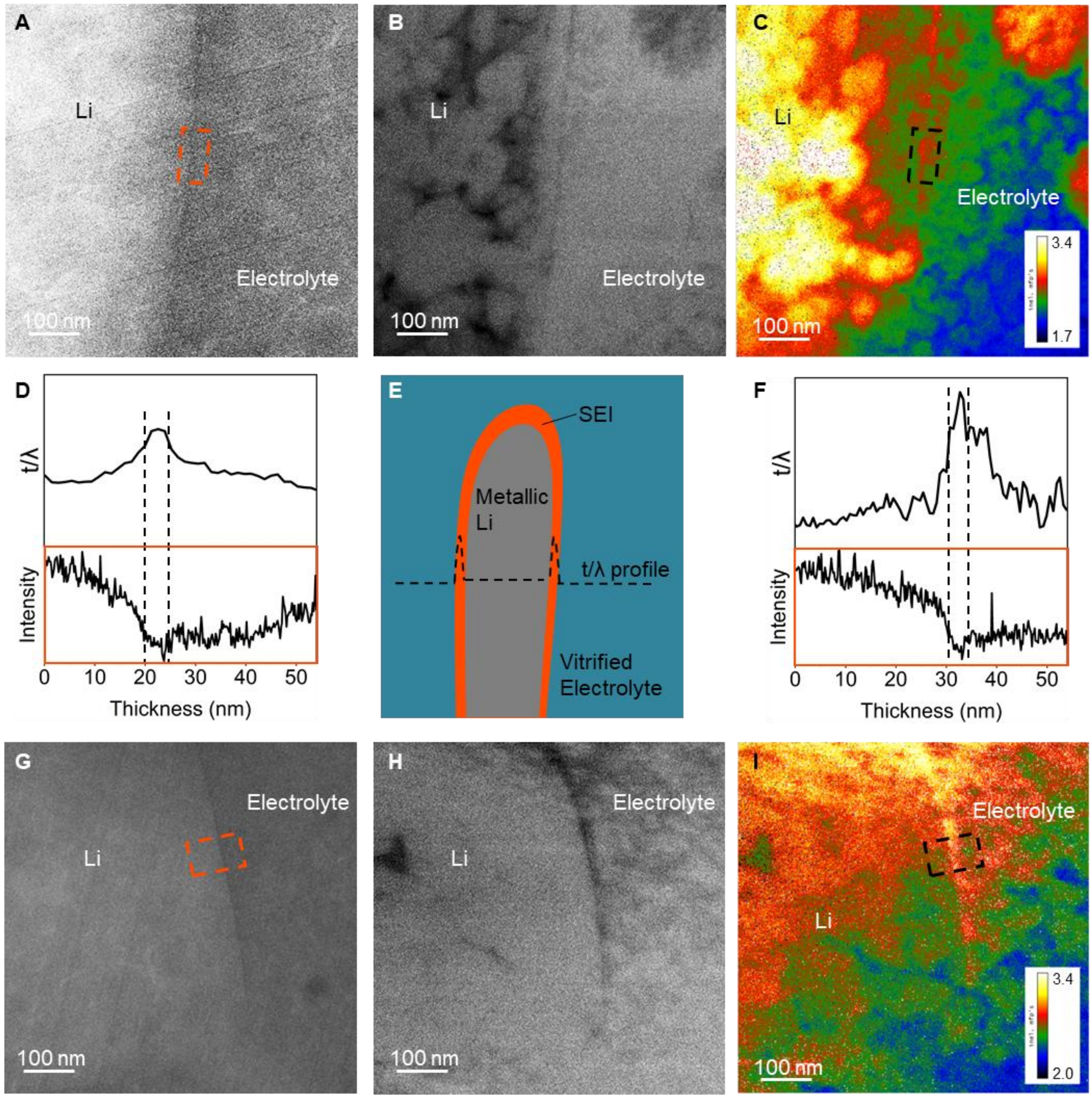

Figure S7. eCryo-EM images and EELS spectrum images of the same Li particles. (**A** and **G**) eCryo-EM images of Li particles at 1 mA cm^{-2} and 0.1 mAh cm^{-2} in 4M LiFSI in DME. (**B** and **H**) Energy filtered EELS spectrum images of the same particles in (**A**) and (**G**), the energy loss range is [45 eV, 75 eV]. (**C)** and (**I**) Relative sample thickness mapping of the same particles in (**A**) and (**G**). (**D**) t/λ profile from the box in (**B**) and line profile from the box in (**A**). (**E**) Schematic

diagram of the sudden increase in t/λ profile. The sudden increase of t/λ coinciding with SEI region is likely attributed to the increase of density in the SEI. (**F**) t/λ profile from the box in (**H**) and line profile from the box in (**G**). The hump of sample thickness fits well to the darker contrast in the eCryo-EM images within SEI regions and the slight contrast difference is due to different mechanisms that contrast is exponential to -t/λ in TEM images (**A**) and (**G**) and is linear to t/λ in thickness mapping (**C)** and (**I**).

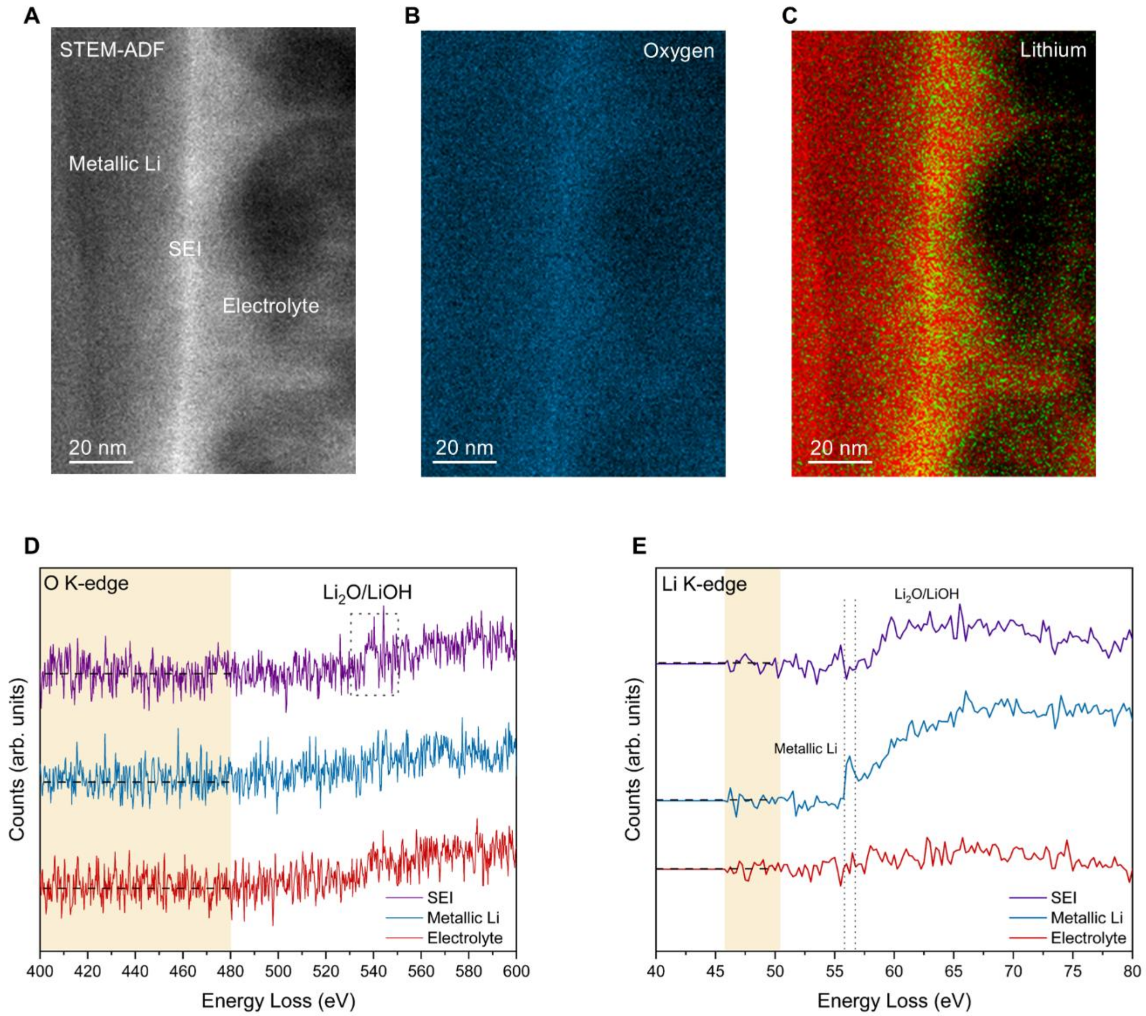

Figure S8. eCryo-EELS data showing chemistry of SEI. (**A**) Annular dark-field (ADF) images of Li particle deposited at 0.5 mAh cm^{-2} and 1 mA cm^{-2} in 4M LiFSI in DME using eCryo sample preparation. (**B**) Spectrum image of the Li particle from the O K-edge region (450 eV to 650 eV). O Kedge. (**C**) Multiple linear least squares (MLLS) fitted image from 45 eV to 85 eV of the Li particle showing distribution of different Li species reference to spectral signature. (**D**) Spectrum image of the Li particle from the O K-edge region (450 eV to 650 eV). O Kedge (**D**) and Li K-edge (**E**) of vitrified electrolyte and different regions of the Li particle. Horizonal dashed lines

represent zero for each profile. Light yellow regions represent the regions for background subtraction. The SEI region is distinct from metallic Li in both O K-edge and Li K-edge. In O K-edge, the SEI region show a ~7.5 eV wide peak supposed to be an overlay of O-containing species such as Li_2O, LiOH, and C=O bonds, which cannot be attributed to an individual species due to the energy resolution. In Li K-edge, there is a distinct peak in the SEI region indicating inorganic species. Combining the EELS spectrum of Li K-edge and O K-edge, we can conclude that the SEI is rich in inorganic species such as Li_2O and LiOH.

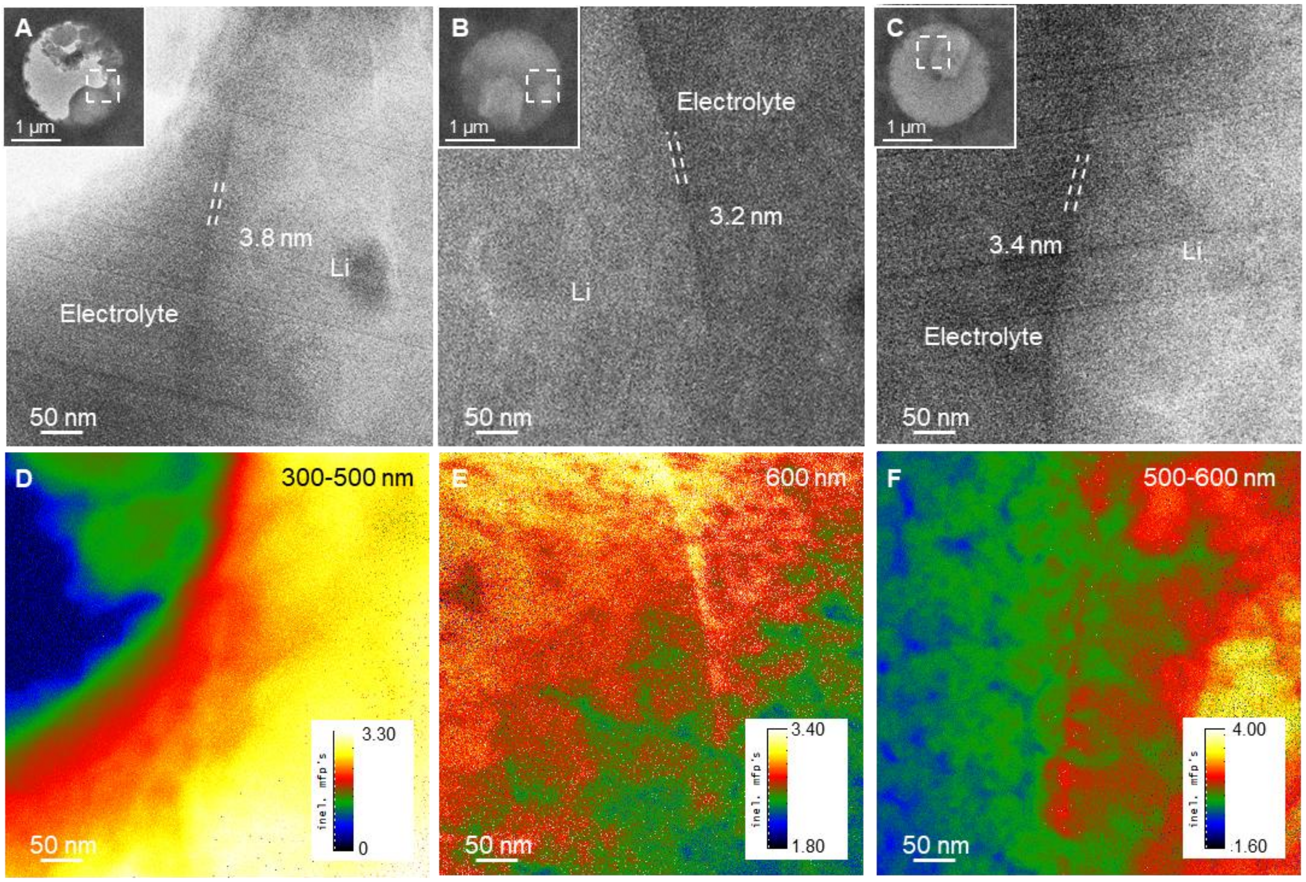

Figure S9. SEI thickness measurement paired with EELS sample thickness mapping. (**A** to **C**) eCryo-EM images of SEI thickness measurement and inset images are the low-mag images of the Li particles at 1 mA cm^{-2} and 0.1 mAh cm^{-2} in 4M LiFSI in DME. Images are rotated to relative to EELS mapping. (**D** to **F**) Relative sample thickness mapping of the Li particles. The mean free path of Li is estimated to be 250 nm, thus the sample thickness is ranged from 300 nm to 600 nm.

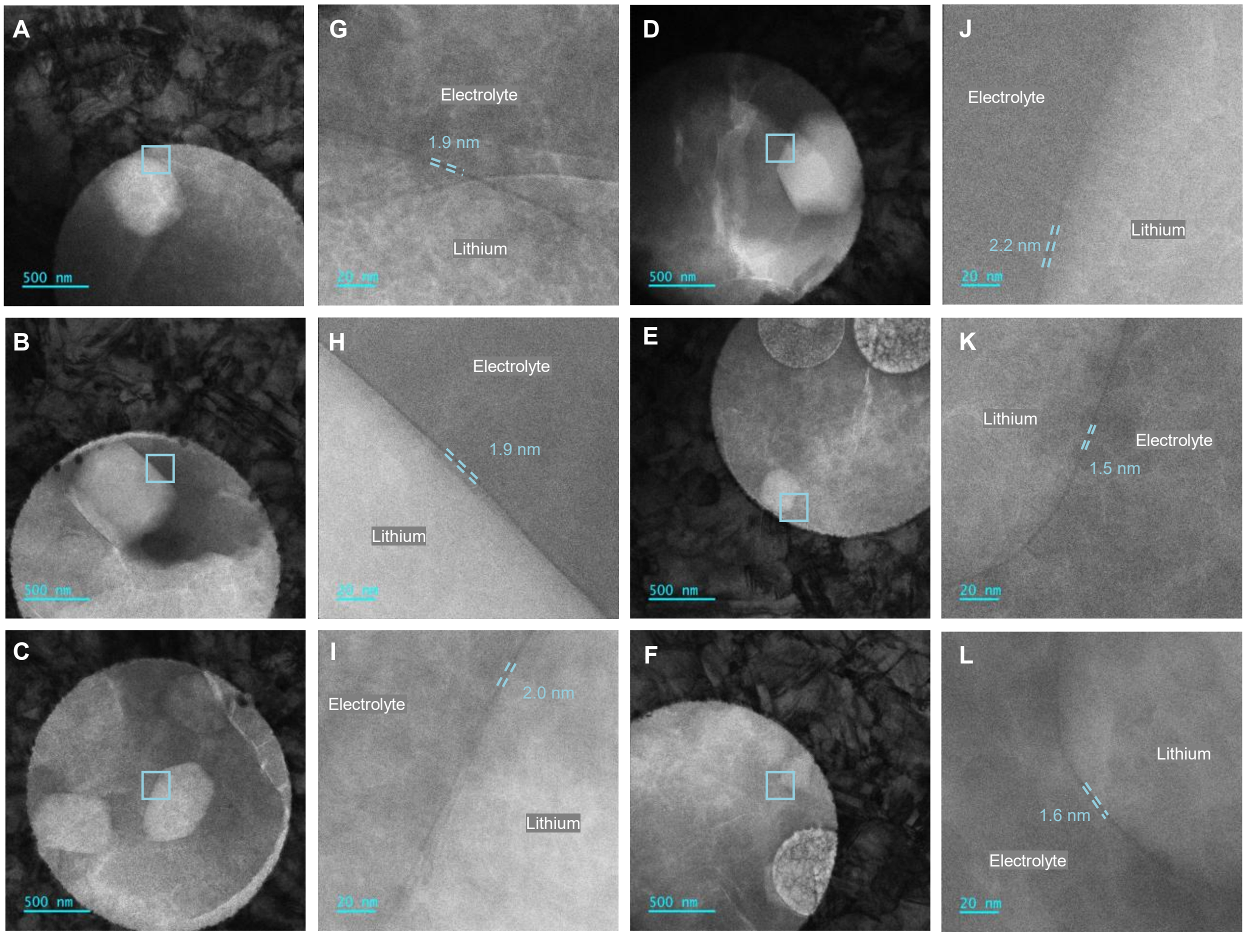

Figure S10. Gallery of eCryo TEM images at 1 mA cm^{-2} and ~0.002 mAh cm^{-2} in 1M LiFSI in DME. (**A** to **F**) Representative low-mag images of lithium particles. (**G** to **L**) Zoomed-in images of the square region left to each image.

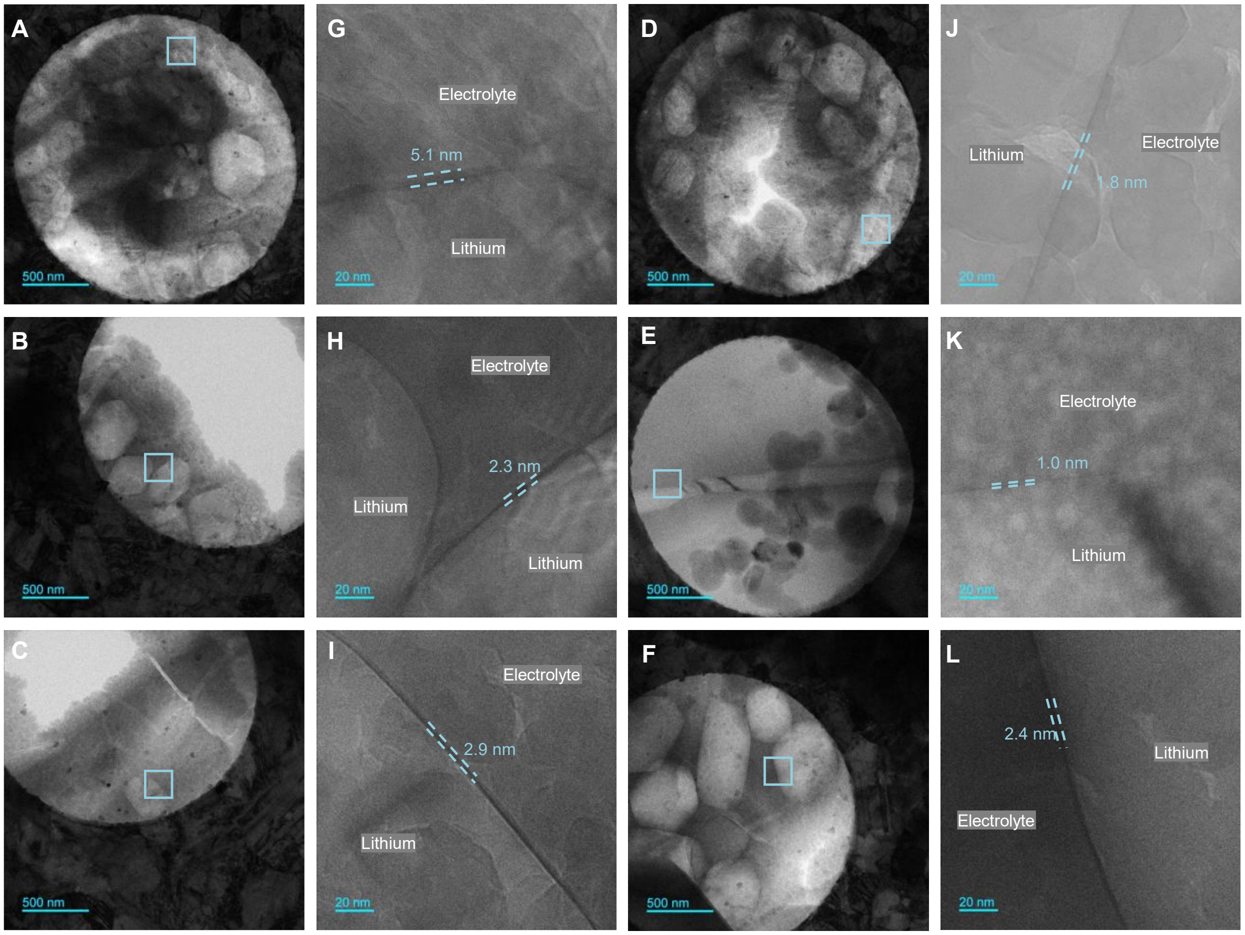

Figure S11. Gallery of eCryo TEM images at 1 mA cm^{-2} and ~0.01 mAh cm^{-2} in 1M LiFSI in DME. (**A** to **F**) Representative low-mag images of lithium particles. (**G** to **L**) Zoomed-in images of the square region left to each image.

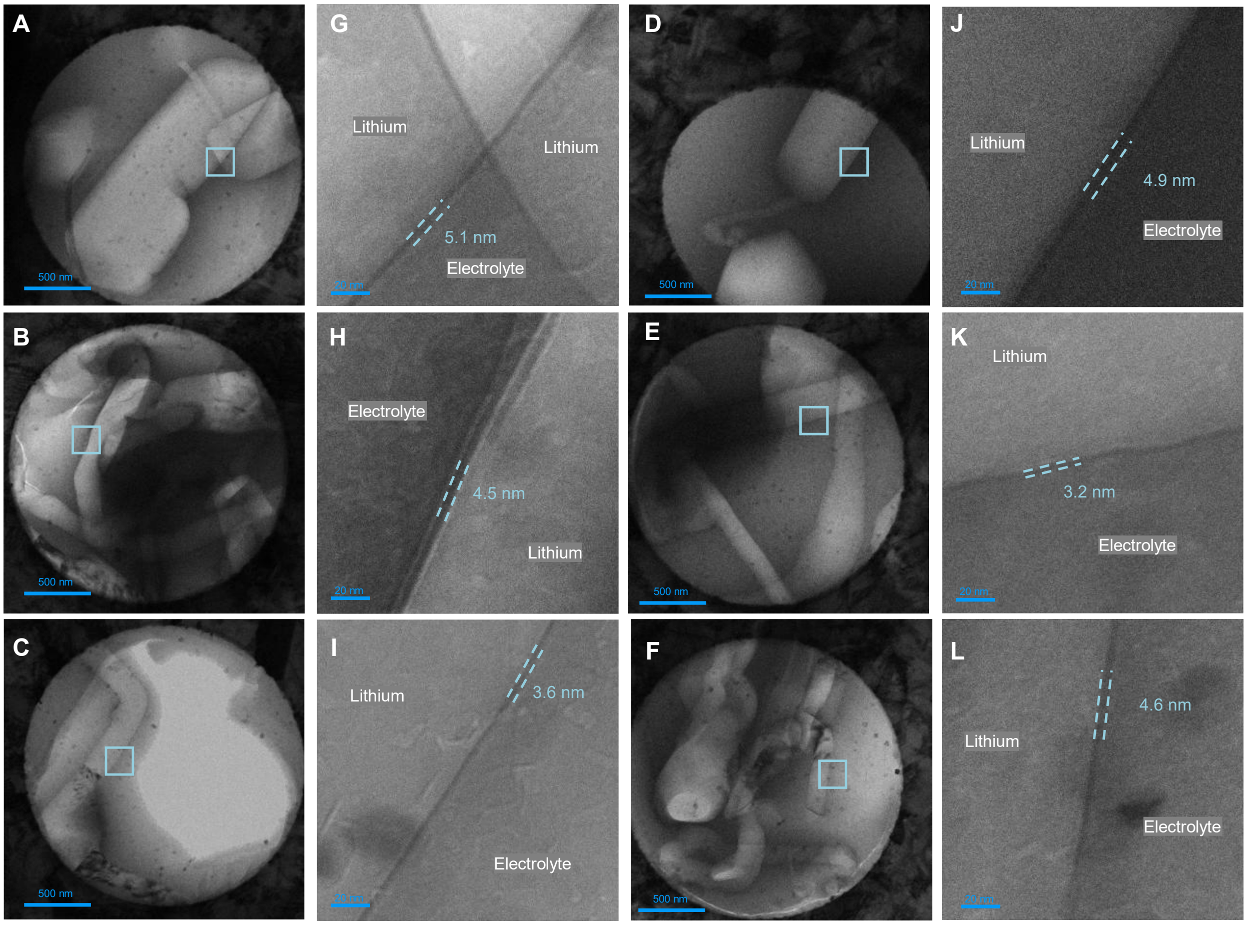

Figure S12. Gallery of eCryo TEM images at 1 mA cm^{-2} and ~0.03 mAh cm^{-2} in 1M LiFSI in DME. (**A** to **F**) Representative low-mag images of lithium particles. (**G** to **L**) Zoomed-in images of the square region left to each image.

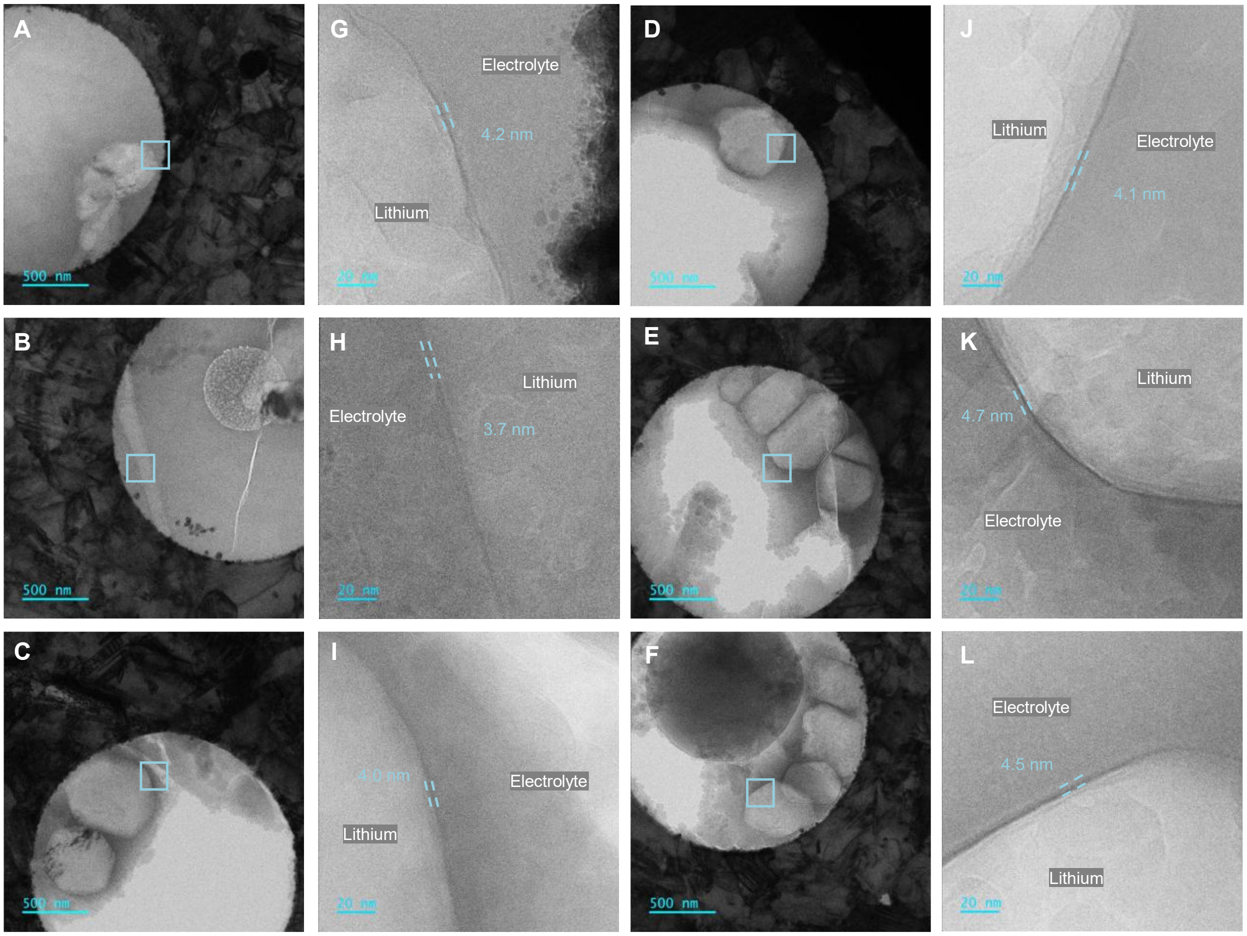

Figure S13. Gallery of eCryo TEM images at 1 mA cm^{-2} and ~0.06 mAh cm^{-2} in 1M LiFSI in DME. (**A** to **F**) Representative low-mag images of lithium particles. (**G** to **L**) Zoomed-in images of the square region left to each image.

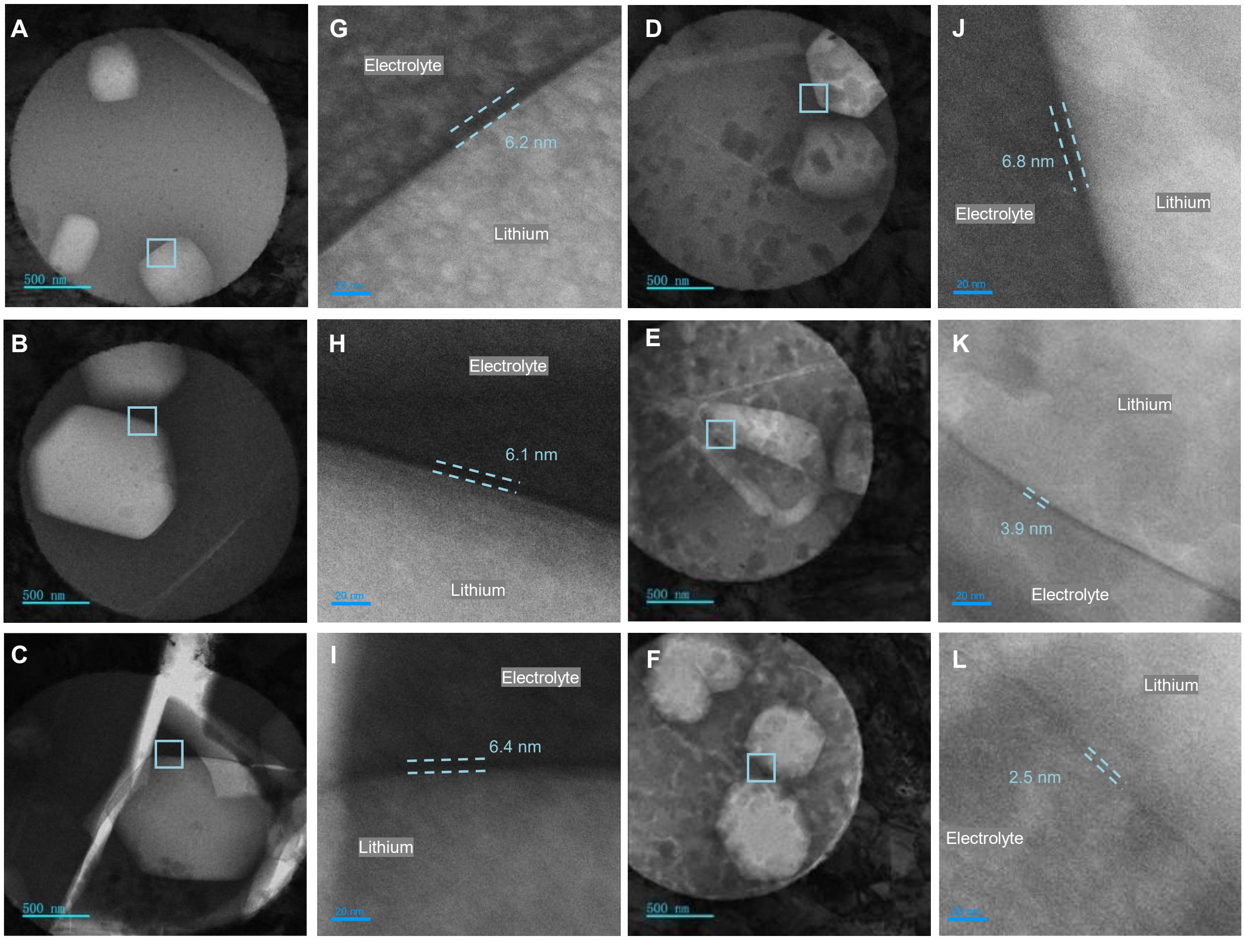

Figure S14. Gallery of eCryo TEM images at 1 mA cm^{-2} and ~0.10 mAh cm^{-2} in 1M LiFSI in DME. (**A** to **F**) Representative low-mag images of lithium particles. (**G** to **L**) Zoomed-in images of the square region left to each image.

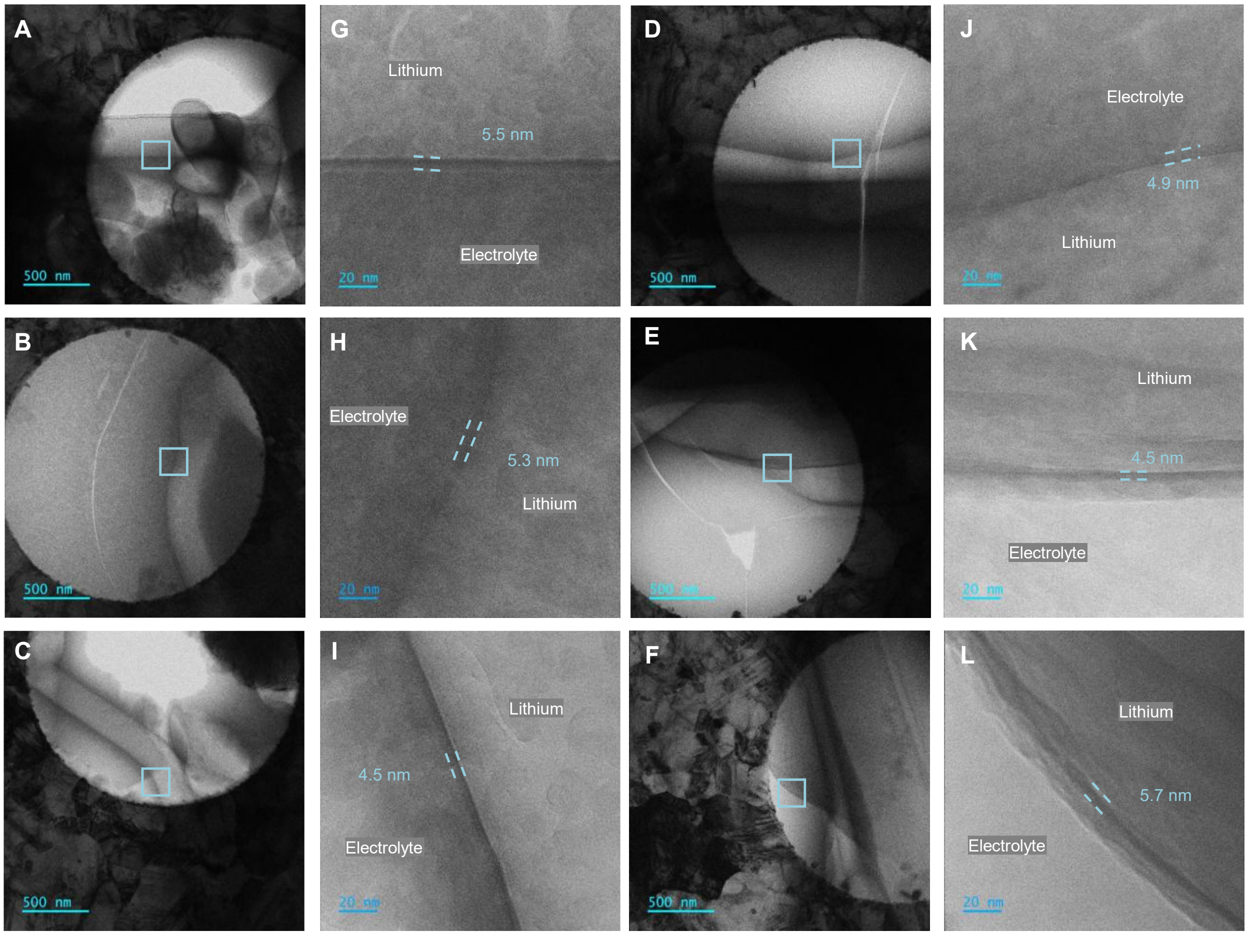

Figure S15. Gallery of eCryo TEM images at 1 mA cm^{-2} and ~0.20 mAh cm^{-2} in 1M LiFSI in DME. (**A** to **F**) Representative low-mag images of lithium particles. (**G** to **L**) Zoomed-in images of the square region left to each image.

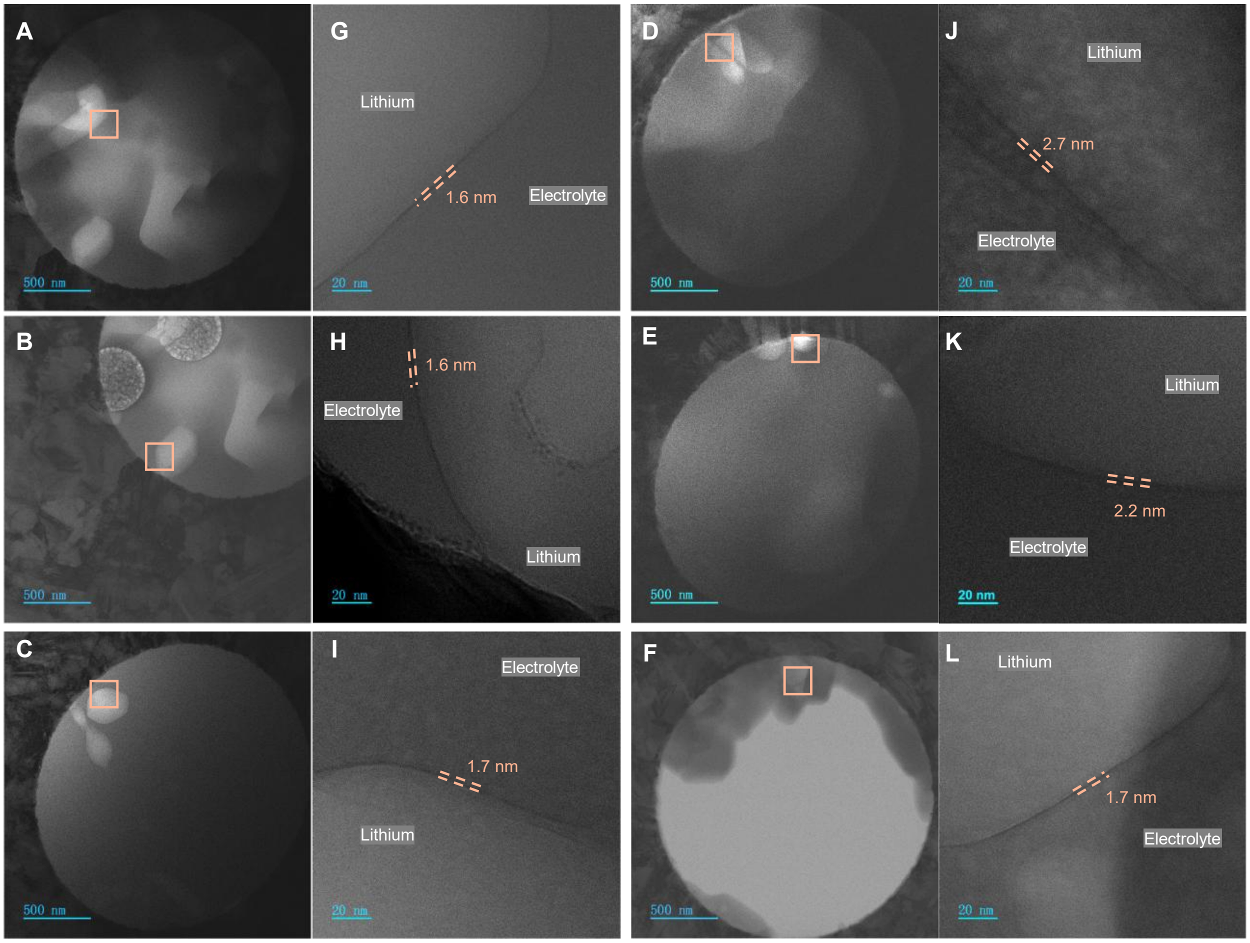

Figure S16. Gallery of eCryo TEM images at 1 mA cm^{-2} and ~0.002 mAh cm^{-2} in 4M LiFSI in DME. (**A** to **F**) Representative low-mag images of lithium particles. (**G** to **L**) Zoomed-in images of the square region left to each image.

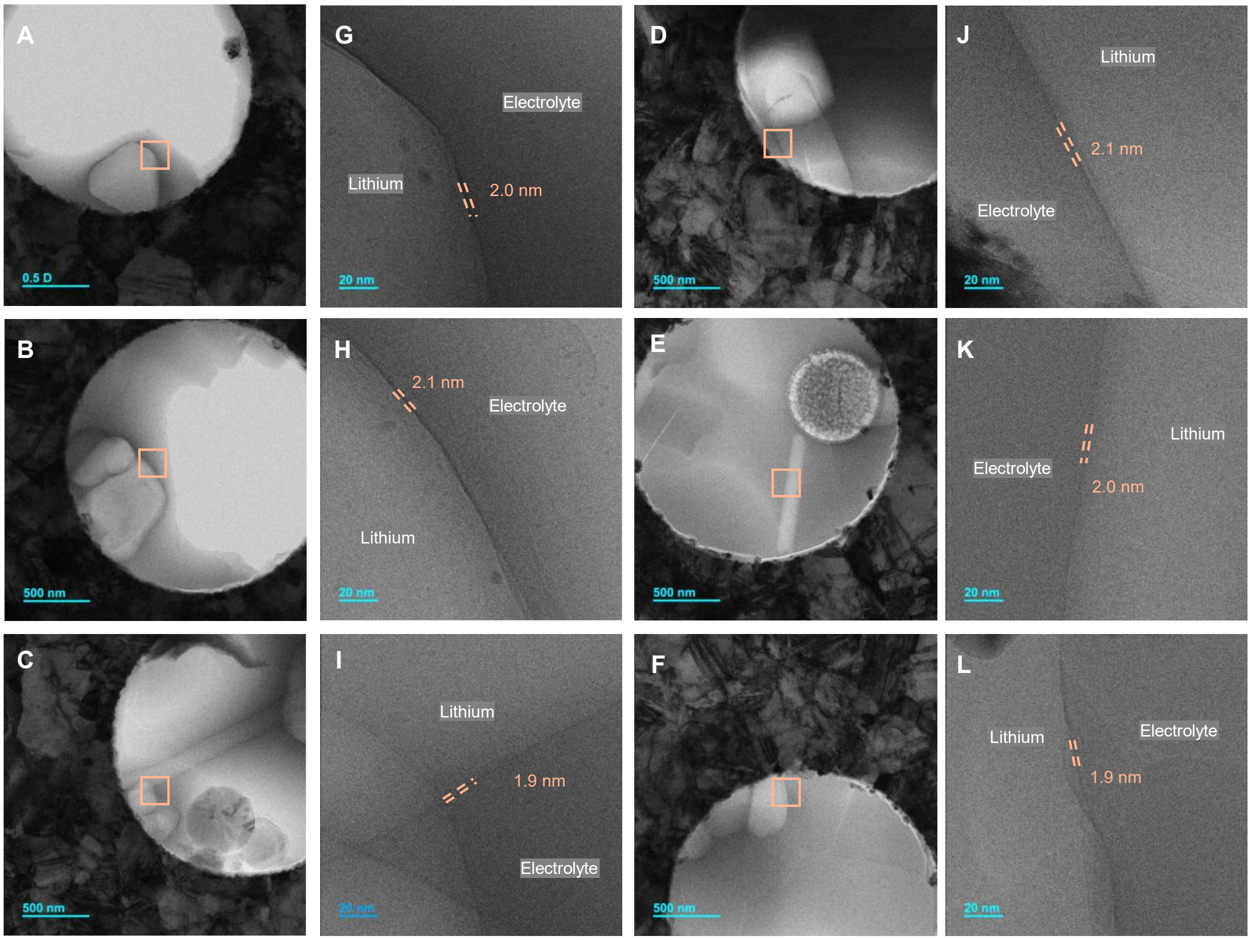

Fig. S17. Gallery of eCryo TEM images at 1 mA cm^{-2} and ~0.01 mAh cm^{-2} in 4M LiFSI in DME. (**A** to **F**) Representative low-mag images of lithium particles. (**G** to **L**) Zoomed-in images of the square region left to each image.

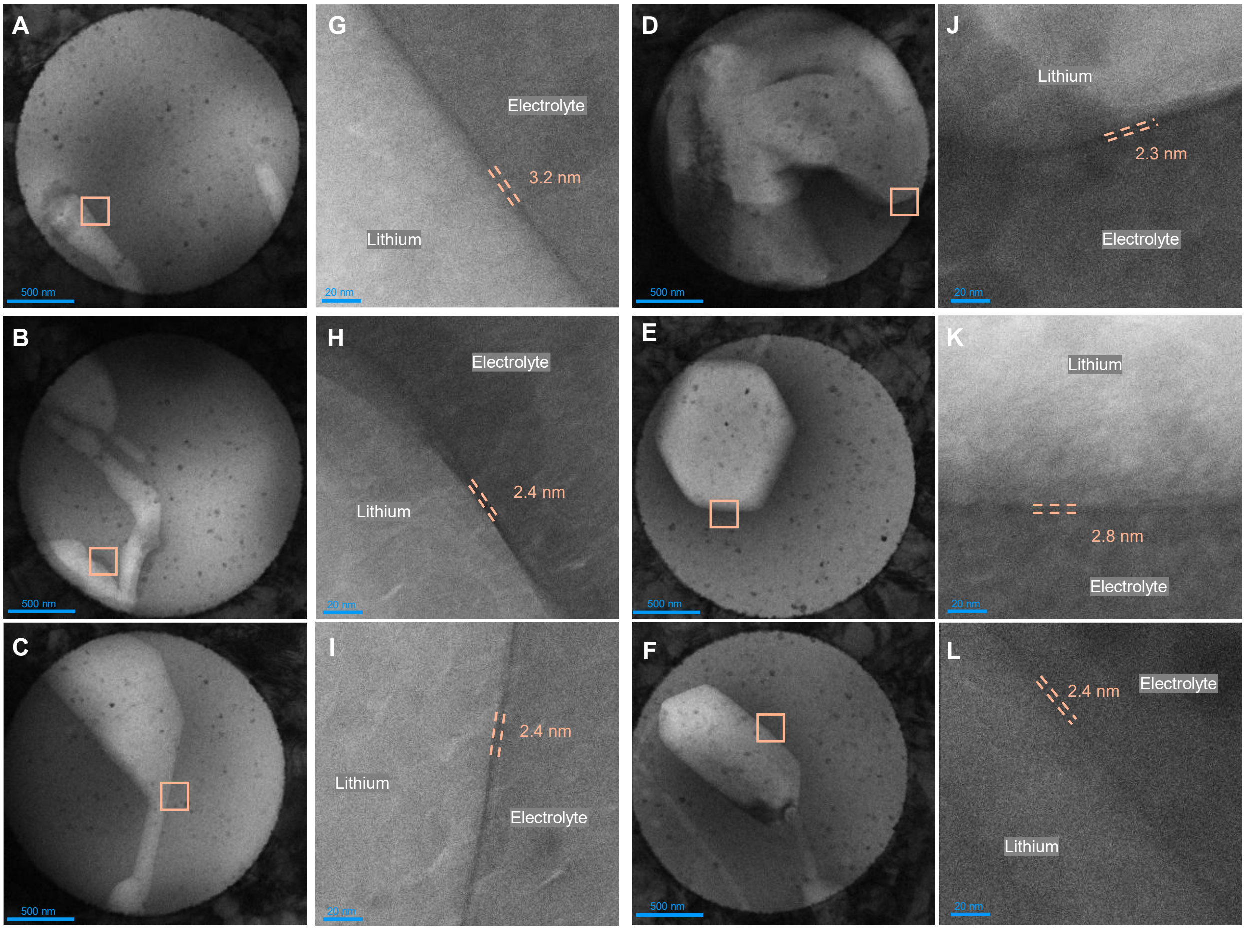

Fig. S18. Gallery of eCryo TEM images at 1 mA cm^{-2} and ~0.03 mAh cm^{-2} in 4M LiFSI in DME. (**A** to **F**) Representative low-mag images of lithium particles. (**G** to **L**) Zoomed-in images of the square region left to each image.

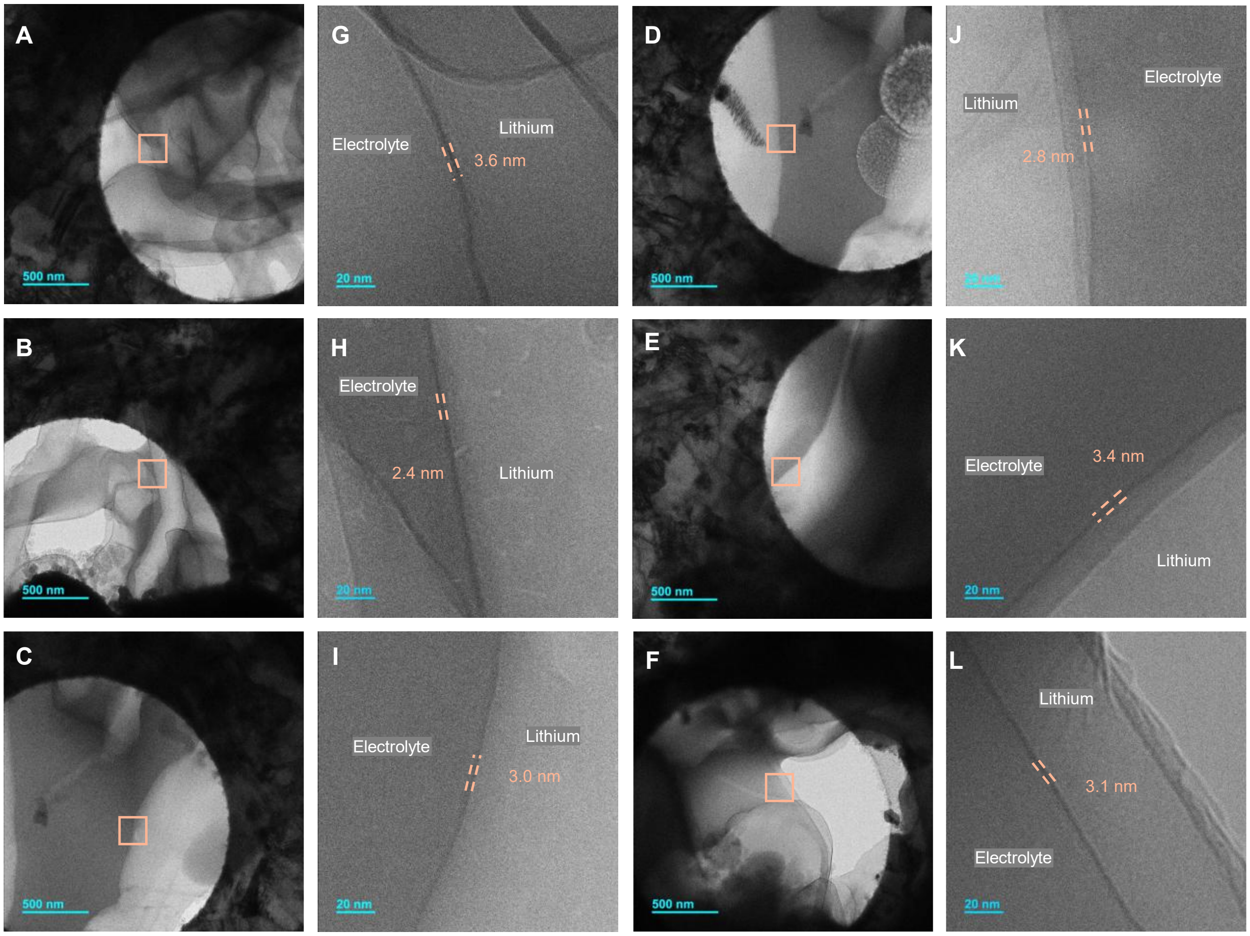

Fig. S19. Gallery of eCryo TEM images at 1 mA cm^{-2} and ~0.06 mAh cm^{-2} in 4M LiFSI in DME. (**A** to **F**) Representative low-mag images of lithium particles. (**G** to **L**) Zoomed-in images of the square region left to each image.

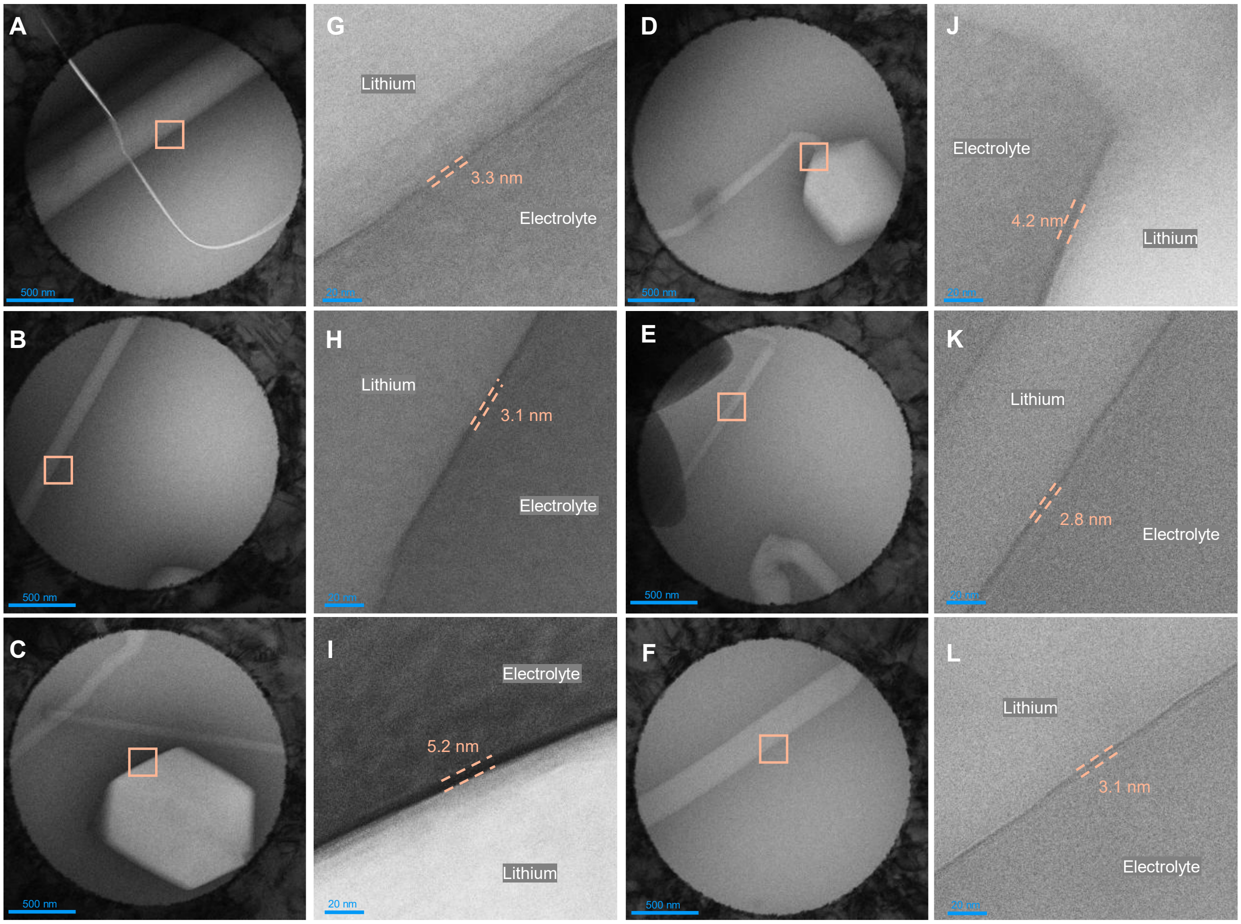

Fig. S20. Gallery of eCryo TEM images at 1 mA cm^{-2} and ~0.10 mAh cm^{-2} in 4M LiFSI in DME. (**A** to **F**) Representative low-mag images of lithium particles. (**G** to **L**) Zoomed-in images of the square region left to each image.

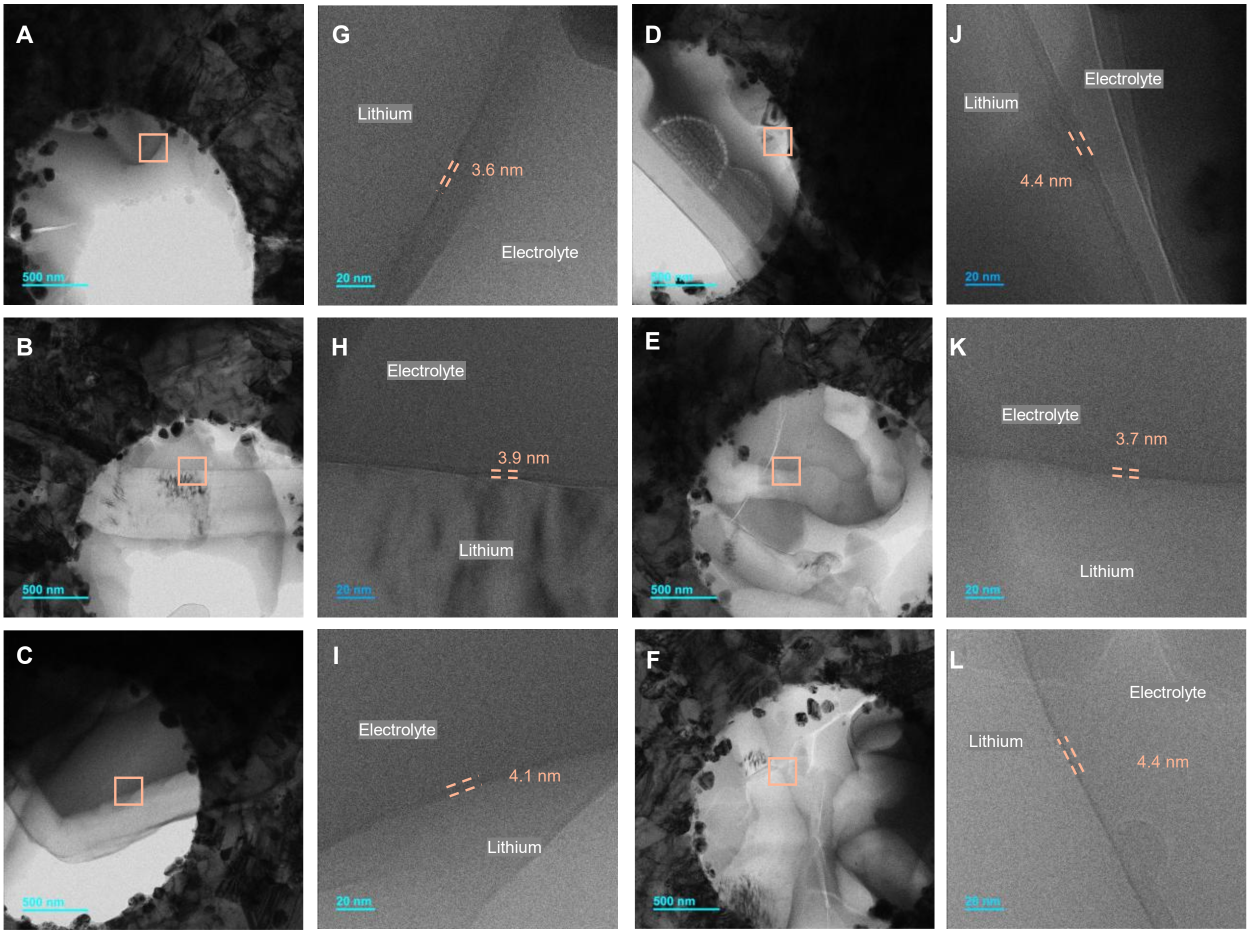

Fig. S21. Gallery of eCryo TEM images at 1 mA cm^{-2} and ~0.20 mAh cm^{-2} in 4M LiFSI in DME. (**A** to **F**) Representative low-mag images of lithium particles. (**G** to **L**) Zoomed-in images of the square region left to each image.

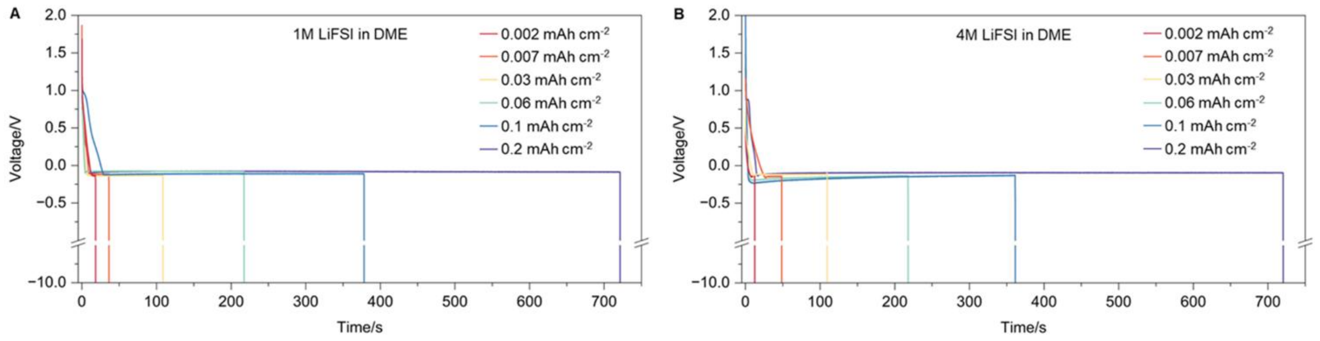

Fig. S22. Voltage profiles for all the data points shown in Figure 4G. (**A**) 1M LiFSI in DME. (**B**) 4M LiFSI in DME.

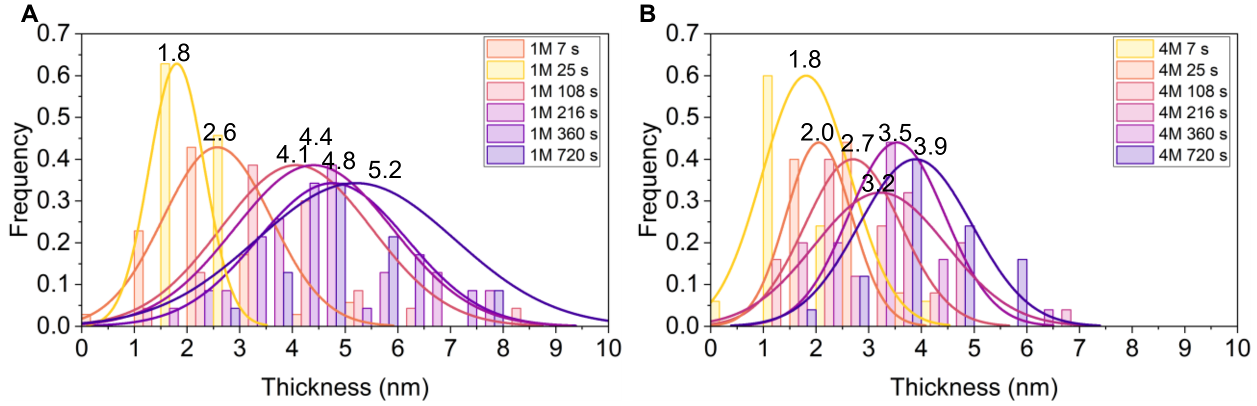

Fig. S23. Distribution of SEI thickness under different deposition time. (**A**) Distribution of SEI thickness in 1M LiFSI in DME. (**B**) Distribution of SEI thickness in 4M LiFSI in DME.

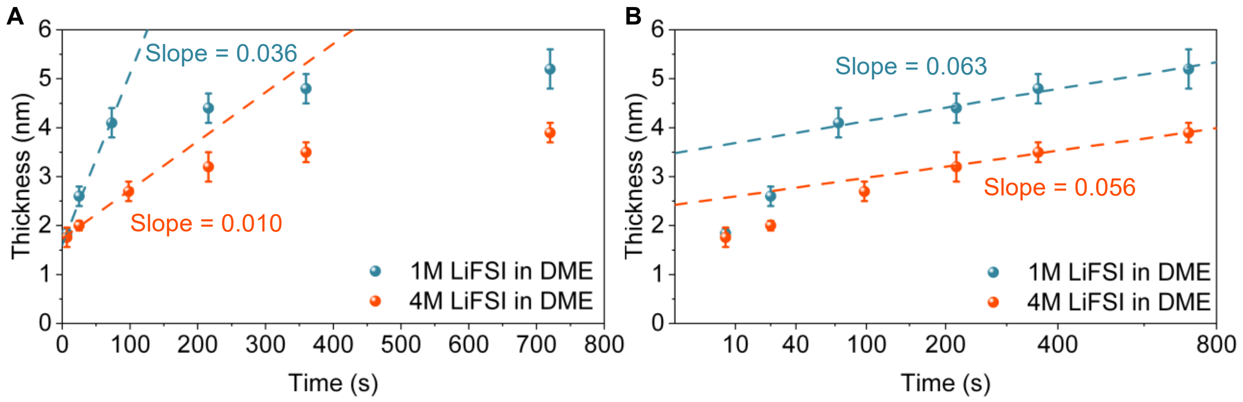

Figure S24. Early-stage SEI growth kinetics analysis. (**A**) SEI thickness profile plotted versus a linear x-axis. The first three data points appear linear with time, indicating a reaction-limited growth regime. (**B**) SEI thickness profile plotted versus the square root of time. The last three data points appear linear to the square root of time, indicating a diffusion-limited growth mechanism. The slopes for both the high performance and conventional electrolyte are within 10% of each other.
